# The Co-Administration of Fluoroquinolones Strongly Increases the Anticancer Efficacy of Carboplatin Treatment—Novel Insights for Breast Cancer Chemotherapy from the Canine Mammary Tumor Model

**DOI:** 10.3390/biology15080604

**Published:** 2026-04-11

**Authors:** Michele Tomanelli, Lorella Maniscalco, Katia Varello, Chaimae Sellak, Isabella Martini, Tullio Florio, Paola Modesto, Aldo Pagano

**Affiliations:** 1 Department of Experimental Medicine, University of Genoa, 16132 Genova, Italy; micheletomanelli1988@gmail.com (M.T.); chaimae.israe@hotmail.com (C.S.); 2 Istituto Zooprofilattico Sperimentale del Piemonte, Liguria e Valle D’Aosta, 10154 Turin, Italy; lorella.maniscalco@unito.it (L.M.); isabella.martini@izsplv.it (I.M.); 3 Department of Veterinary Science, University of Turin, 10095 Grugliasco, Italy; 4 Department of Internal Medicine (DIMI), University of Genoa, 16132 Genova, Italy; tullio.florio@unige.it; 5 IRCCS AOM Ospedale Policlinico San Martino, 16132 Genoa, Italy

**Keywords:** canine mammary tumors, comparative oncology, chemotherapy, drug repositioning, One Health, breast cancer

## Abstract

Human breast cancer (HBC) is the most common and often lethal malignancy in women. Canine mammary tumors (CMTs) share significant molecular and clinical characteristics with HBC, which makes dogs a valuable spontaneous model for the study of HBC. HBC chemotherapy treatment relies mainly on carboplatin, which is effective but, in turn, highly toxic. Here we tested enrofloxacin, a Minichromosomal Maintenance Complex Component (MCM2) inhibitor, for its ability to increase tumor cell sensitivity to platinum-based drugs, thus suggesting a potential synergistic therapeutic strategy. CMT samples were used to establish primary cell cultures. Cells were treated with carboplatin, enrofloxacin, and their combination at different concentrations. Cytotoxic and antiproliferative effects were assessed using xCELLigence and MTT assays. Single-drug treatments exert limited effects on cell proliferation, while enrofloxacin significantly enhances carboplatin efficacy, leading to a complete growth arrest within 48 h. The MTT assay confirms a strong synergistic effect of the two drugs, whereas the Dose Reduction Index analysis indicates that carboplatin could be decreased without losing effectiveness. These findings suggest that combined therapy could represent a more effective and less toxic option for HBC and CMTs. This work also strengthens the possible use of the canine model for cancer studies within a One Health framework.

## Simple Summary

Breast cancer is a disease that behaves comparably in women and female dogs, making dogs a useful natural model for studying new treatments that could also help humans. In dogs, surgery is the major treatment for mammary tumors. However, many animals still experience relapse of the disease for which the drug carboplatin is often used. This drug can be effective but it also causes relevant side effects. In this study we report that the co-administration of the antibiotic enrofloxacin can synergize with carboplatin, allowing its dose to be lowered. Canine cancer cells were grown and tested for their susceptibility. We grew tumor cells taken directly from dogs and tested carboplatin, enrofloxacin, or their combined administration. We found that enrofloxacin greatly increases the ability of carboplatin to impede or halt the proliferation of tumor cells. We show that their combined treatment is effective even at low dosages of carboplatin, suggesting the possibility of a therapy that is more powerful and less toxic. These results may help improve cancer care for dogs, reduce treatment costs and risks, and provide new insights that could be relevant for future breast cancer therapies in humans.

## 1. Introduction

Human breast cancer (HBC) is the most commonly diagnosed tumor in women, representing the second major cause of cancer-related death after lung cancer [1]. However, the introduction of new drugs and the advancement in diagnostic techniques have significantly improved overall survival in the past 20 years [2]. The human Elston and Ellis grading method is commonly used to classify HBC considering tubular formation, nuclear pleomorphism, and mitotic count [3]. This method strongly correlates with the prognosis. Interestingly, for several years, the method was also adopted with canine mammary tumors (CMTs) due to the widespread similarities between the two malignancies [4,5,6,7]. In 2019 the CMT classification system was updated by the WHO and the Davis-Thompson DVM Foundation [8,9].

CMTs are the most frequent cancers in sexually intact female dogs, accounting for almost 50% of canine neoplasms [8,9,10]. They share with HBCs many epidemiological, biological, clinicopathological and genetic features [11,12,13,14]. The indications supporting the validity of the dog as a model for the study of breast cancer lie primarily in the following aspects: (1) a shared spontaneous development, (2) a similar age and hormonal etiology, (3) shared risk factors and (4) a similar disease progression. Also, hormone receptor status, molecular subtypes, metastatic behavior, and response to treatment are very similar [15,16,17,18]. Notably, dogs represent a highly relevant translational model as they develop mammary tumors spontaneously, in contrast to rodent models that require genetic or chemical induction [19,20,21,22,23,24,25,26]. CMTs arise in an intact immune system and in a natural hormonal environment, reflecting the complexity of cancer development in humans [27,28]. Additionally, the shorter lifespan and the more rapid disease progression in dogs allow a faster evaluation of therapeutic interventions [29,30]. In particular, at the molecular level, similarities between CMT and HBC include the dysregulation of key oncogenic pathways and shared biomarkers such as elevated Ki-67 proliferation index, TP53 mutations, altered PTEN expression, and activation of angiogenic factors like VEGF and EGFR, all of which correlate with tumor aggressiveness and reduced survival in both species [31]. Furthermore, basal-like tumors, which largely overlap with the triple-negative breast cancer (TNBC) phenotype, display comparable gene expression profiles between dogs and humans, particularly regarding proliferation-associated genes and reduced luminal marker expression [32].

Taken together, these characteristics make the dog a fruitful model for studying HBC biology and for testing novel diagnostic and therapeutic approaches on spontaneous cancer cells with direct translational relevance to human medicine [14,15,16,17,33,34,35]. In addition, the use of CMTs as HBC model fits perfectly with the One Health approach, as results obtained in animal models are translatable to humans, and vice versa, thus strengthening the possible use of information obtained from individual model organisms about others [36,37].

From a One Health frame perspective, our laboratory aims to reposition in cancer therapy drugs previously approved for the treatment of other pathologies, which brings a significant increase in the efficacy of the combined approach, thanks to synergistic pharmacological effects. In our recent studies on neuroblastoma (NB), we reported the crucial role of the MCM2 gene (a helicase necessary for the replication of tumor cells) in determining an unfavorable prognosis [36,37,38,39,40,41,42,43]. This nuclear action is very well documented in humans, as MCM2 overexpression is considered an extremely sensitive marker of proliferation and malignancy. In dogs, these aspects have been investigated in the case of fibrosarcoma and adenocarcinoma, showing a close and significant correlation between the level of expression of MCM family helicase proteins and the malignancy [40,41,44].

In agreement, other researchers reported a similar role of MCM2 also in breast cancer [45,46,47]. In detail, MCM2 is significantly overexpressed in triple-negative luminal B, triple-negative, and HER2-positive breast cancer [48], which are known for high proliferative rates, compared to other subtypes. MCM2 has been proposed as a marker of high proliferation, high histological grade, and aggressive tumor behavior, often superior to Ki-67 [49]. Moreover, MCM2-targeted strategy treatments have been supposed to succeed in therapy for highly malignant breast cancers such as TNBC [50].

Therefore, the possible pharmacological inhibition of the function/expression of MCM2 might be a promising therapeutic approach. In this context, several inhibitory molecules of the MCM protein family (all belonging to the class of fluoroquinolones) currently used as antibiotics (ciprofloxacin in humans and enrofloxacin in animals) might be considered. The molecular mechanism of inhibition of MCM proteins by enrofloxacin is mainly explained by (1) the direct inhibition of the helicase, (2) the prevention of DNA unwinding during replication, and (3) cell cycle arrest.

Our previous studies in NB mouse models demonstrated that, if co-administered, fluoroquinolones (causing the inhibition of MCM2) and platins (cytotoxic drugs) act synergistically, leading to the strong susceptibility of tumor cells to the combo-therapy [43]. Other studies reported similar results using fluoroquinolones in the treatment of breast cancer cell lines, thus supporting the reposition of fluoroquinolones from antibiotics to anticancer agents [51,52,53,54,55,56].

However, the studies that propose fluoroquinolones as possible new anticancer drugs are currently still based on in vitro and/or preclinical models, and further experiments will be necessary to validate their use in both humans and dogs [54].

The focus of this study is clearly on dogs from a One Health perspective. This is a preclinical, non-mechanistic study with potential for translational development. The hypothesis is to identify adjuvant therapies that enhance the efficacy of low-dose cytotoxic drugs, reducing their cytotoxic effects while maintaining the same anticancer activity.

Therefore, this work demonstrates that the co-administration of these two drugs might have several positive consequences: (1) a strong increase in drug potency and efficacy [43], (2) the possibility of treating relapsing patients who are resistant to doses of the drug normally used, and (3) the ability to maintain drug efficacy at lower doses, thus reducing the toxicity of the therapy.

## 2. Materials and Methods

### 2.1. Histological Analysis

CMT samples were surgically removed, fixed in 10% buffered formalin and sent to Istituto Zooprofilattico Sperimentale del Piemonte, Liguria E Valle D’Aosta, Turin, Italy. The samples were processed and embedded in paraffin. Then, 4 ± 2 μm sections were obtained with a microtome and stained with hematoxylin–eosin. The stained samples are classified following the WHO and CL Davis Foundation Criteria of Canine Mammary Tumors [9], and the histological grading is assigned according to the up-to-date criteria present in the literature and internationally shared [7,57].

### 2.2. Primary Cell Culture Protocols

CMTs were received from veterinary clinics located in Savona and Sestri Levante (n = 14) and the University of Perugia (n = 3).

Samples were collected over a period of 18 months. A total of 33 mammary tumors were identified. No selection was made during sample collection. The number of bitches was the same as that of the cases (only primary tumors were collected). All samples received were processed. Only those for which a cell culture could be established were included in this study. However, we did not receive any grade III tumors from the veterinary clinics involved, which we intend to investigate in a future study. The solid masses were minced in about 1 mm diameter pieces, washed with Dulbecco’s Phosphate-Buffered Saline (PBS) (EuroClone, Milan, Italy) and filtered with 0.32 μM diameter filters (EuroClone, Milan, Italy) to reduce the presence of bacterial and other contaminants. The cells were then recovered from the filter using PBS, and the resulting solutions were centrifugated at 1200 RPM for 10 min and the supernatant removed. Pellets were resuspended in Dulbecco’s Modified Essential Medium (DMEM), Fetal Bovine Serum (FBS) (10%; EuroClone, Milan, Italy), L-glutamine (2 mM; EuroClone, Milan, Italy), Penicillin–Streptomycin (100 U/mL/100 μg/mL; EuroClone, Milan, Italy), and Amphotericin B (250 μg/mL; Sigma Aldrich, St. Louis, MO, USA) and plated in T-25 cm^2^ flasks (Sarstedt Ag & Co. KG, Nümbrecht, Germany). Considering that fibroblasts detach earlier than epithelial cells, we treated cells with trypsin and observed them under the microscope, confirming the preferential removal of fibroblasts. Trypsin was then aspirated to remove fibroblasts, and the remaining adherent cells were treated again with trypsin until detachment. Trypsin was subsequently inactivated and the cells were plated. These steps were repeated for 4–5 weeks until the complete disappearance of fibroblasts. Over time, only epithelial cells (with a more rounded morphology) remained, eventually filling the entire culture. The cells were maintained in a Heraeus CO_2_ Auto-Zero incubator (Thermo Fisher Scientific, Waltham, MA, USA) at 37 °C in a humidified atmosphere of 5% (*v*/*v*) CO_2_ in air and routinely subcultured twice weekly.

### 2.3. xCELLigence System Cytotoxicity Assays

Primary mammary tumor cultures were seeded in 16-well E-Plate view support (Acea Biosciences, San Diego, CA, USA) at 3000 cells/well concentrations. On the second day, the cells were treated with carboplatin, enrofloxacin and their combination. Pharmacokinetic studies in dogs report plasma Cmax values of approximately 2–5 µg/mL after therapeutic dosing of enrofloxacin, corresponding to about 5–14 µM. The concentrations tested in vitro (30–1000 µM) therefore represent approximately 3–100× the physiological plasma concentration. The small number of samples used in the pilot experiment were treated with the following concentrations: carboplatin (10, 30, 100, and 300 µM), enrofloxacin (30, 100, 300, and 1000 µM) and their combination (10 µM + 30 µM, 30 µM + 100 µM, 100 µM + 300 µM, and 300 µM + 1000 µM). A larger number of samples was treated with carboplatin (300 µM), enrofloxacin (400 µM) and their combination (300 µM + 400 µM). These concentrations were determined after a previous analysis with an increasing scale of concentrations. The effects of the treatments on proliferation were monitored for 120 h at intervals of 30 min. The assay was performed in real time with the xCELLigence RTCA MP System (Agilent technologies, Santa Clara, CA, USA) [58,59]. The system used ( xCELLigence) is specifically designed to highlight in real time the slowing down and/or arrest of the cell cycle. The plates used for the seed integrate a gold microelectrode at the bottom. Cell cytotoxicity was expressed as an arbitrary unit called Cell Index (CI), which is a parameter generated by the xCELLigence RTCA MP system and is calculated from relative changes in electrical impedance. Variations in CI over time quantitatively represent cellular behavior (cell number, viability, morphology, and adhesion) based on the degree to which cells interact with and modulate the electrical impedance measured by microelectrodes integrated into the culture plates. The experiments were conducted following the manufacturer’s protocol. A real-time proliferation assay was conducted for 160 h after the treatments.

### 2.4. MTT Cytotoxicity Assay

Primary cultures derived from CMTs were seeded in 96-well plates at a density of 3000 cells/well in complete growth medium and allowed to adhere overnight. On the following day, cells were treated with carboplatin (10, 30, 100, and 300 µM), enrofloxacin (30, 100, 300, and 1000 µM), and the combination of both drugs (10 µM + 30 µM, 30 µM + 100 µM, 100 µM + 300 µM, and 300 µM + 1000 µM). Cell viability was evaluated after 72 h of treatment using the MTT assay (3-(4,5-dimethylthiazol-2-yl)-2,5-diphenyltetrazolium bromide), following standard protocols. At the end of the treatment period, MTT reagent was added to each well and incubated for 3–4 h at 37 °C. The resulting formazan crystals, produced by metabolically active cells, were solubilized using DMSO, and absorbance was measured at 570 nm with a microplate reader. The absorbance values were used to calculate cell viability as a percentage relative to untreated control cells. All treatments were performed in triplicate, and data were expressed as mean +/− standard deviation.

### 2.5. Statistical Analysis

Statistical significance between drug responses was calculated using two-way ANOVA. The Bonferroni test was used to perform multiple comparisons. *p*-values less than 0.05 were considered statistically significant. The statistical calculations were performed using Graph-pad Prism version 9 (GraphPad Software, La Jolla, CA, USA).

## 3. Results

### 3.1. CMTs Exhibit Different Histological Subtypes

The tissue samples were histologically classified using hematoxylin–eosin staining, to assess cellular and tissue features. As summarized in Table 1, 6 out of the 17 CMTs analyzed were identified as benign tumors. The remaining 11 samples were classified as malignant: 7 Grade I and 4 Grade II carcinomas.

### 3.2. Enrofloxacin Potentiates the Antiproliferative Activity of Carboplatin in CMTs

To evaluate the increase in antiproliferative activity of carboplatin when co-administered with enrofloxacin, we first treated primary cell cultures of three CMTs with different concentrations of carboplatin (10, 30, 100, and 300 µM) or enrofloxacin (30, 100, 300, and 1000 µM) individually, in order to identify conditions and treatment duration beyond which effects are evident with the single drugs.

The results demonstrated that no effects on proliferation are detectable up to 150 h of treatment with the single drugs, at any dosage used. The first visible effects are detectable after 150 h with at least 300 µM carboplatin and 300 µM enrofloxacin individually administered.

**Figure 1:**
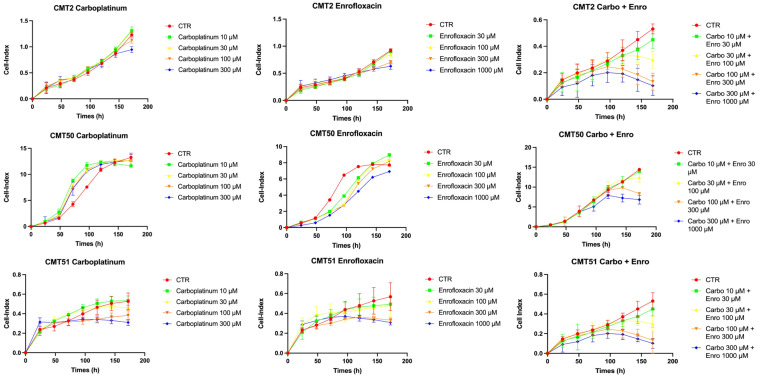
Reduction in proliferation index in CMT primary cells. The Cell Index represents an arbitrary measure of cellular behavior (cell number, viability, morphology, and adhesion). The three CMTs were tested in triplicate and analyzed individually. Two-way ANOVA reported significant differences between the combo carboplatin 300µM + enrofloxacin 1000 µM and carboplatin alone 300 µM. CTR: untreated control cells.

On the contrary, combined treatment with the two drugs produced inhibitory effects on proliferation at concentrations of 100 µM for carboplatin and 300 µM for enrofloxacin. starting after 100 h of treatment (Figure 1).

### 3.3. Enrofloxacin Increases the Antiproliferative Effect of Carboplatin in an Expanded Set of CMTs

**Figure 2:**
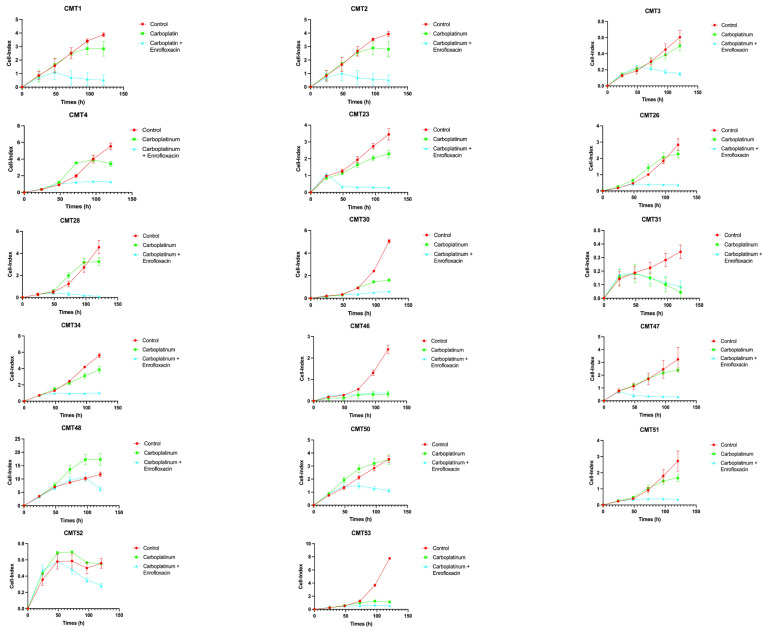
The CMT primary cell proliferation index is reduced by the combination of carboplatin and enrofloxacin with respect to the control. The Cell Index represents an arbitrary measure of cell status in culture, reflecting cell number and viability. Every experiment was performed in triplicate and analyzed individually.

**Figure 3:**
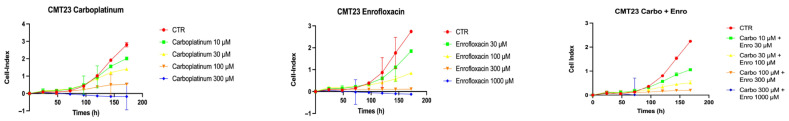
Effects on cell proliferation in TM23 benign tumor samples. The Cell-index represents an arbitrary measure of cell status in culture, reflecting cell number and viability.

Following the results obtained from single-drug treatments, we tested the combination of the two drugs to assess the possible improvement in efficacy in distinct subtypes of tumors characterized by different grades of malignancy. We treated tumor cells with two intermediate doses of carboplatin and/or enrofloxacin to be compared to the use of the single drugs. We treated 17 CMTs whose proliferation before and after the treatments was monitored with xCELLigence technology [59,60,61]. At a concentration of 300 µM, carboplatin did not exert any significant antiproliferative effect in 14 of the 17 tumors examined in the first 48–72 h, whereas it began to counteract proliferation after this lag of time. On the contrary, with the co-administration of a non-toxic concentration of enrofloxacin (400 µM), the effect became evident in the first hours of treatment in all the samples, leading to a total proliferative arrest already after 48 h, although, in two samples (CMT 31 and CMT 53), carboplatin already exerted its antiproliferative effect in the first 48 h even in the absence of enrofloxacin. Interestingly, in the case of these benign tumors, the response to carboplatin and enrofloxacin treatments, both individually and in combination, resulted in a comparable effect, suggesting that the synergistic action between the two drugs in inhibiting cell proliferation is particularly effective in the case of malignancy and that this synergy possibly also influences other aspects of tumor growth (Figure 2 and Figure 3). When using chemotherapy in humans, but also in dogs, multiple drug resistance may develop due to the function of an efflux pump called P-glycoprotein (MDR1); benign tumors themselves express low levels of MDRs and as such are susceptible to even lower doses of chemotherapy [40].

**Figure 4:**
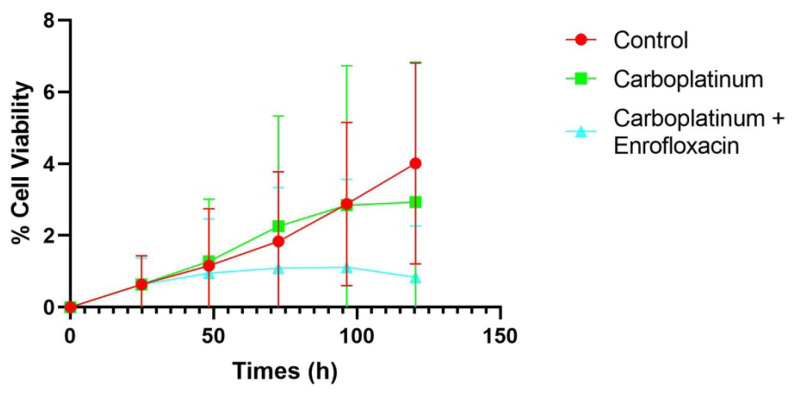
The mean cell proliferation index of all drug-treated CMT primary cell lines. Treatment with the combination of carboplatin and enrofloxacin resulted in a decreased proliferation rate compared to the control. Data represent the average of all experiments performed in triplicate.

Altogether, these results demonstrate a significant enhancement of carboplatin’s antiproliferative/cytotoxic effect when co-administered with enrofloxacin in several types of CMTs, with potentially important clinical implications. The average of all samples tested is shown in Figure 4.

### 3.4. Carboplatin and Enrofloxacin Act Synergistically Exerting a Combined Antiproliferative Effect

To determine the possible synergy between carboplatin and enrofloxacin treatment, we determined their cytotoxic effect on 10 tumor primary cell cultures (TM1, TM2, TM3, TM4, TM23, TM26, TM30, TM47, TM50, and TM51) To this aim we took advantage of the MTT cell viability between the two drugs in inhibiting cell proliferation. The inhibition resulted particularly effective in the case of malignancy and this synergy probably also influences other aspects of tumor growth. Cells were treated with four different concentrations of carboplatin (10 µM, 30 µM, 100 µM and 300 µM), in combination with four different concentrations of enrofloxacin (30 µM, 100 µM, 300 µM, and 1000 µM). The concentrations of the two drugs used were in a ratio of approximately 1:3 (10 µM + 30 µM, 30 µM + 100 µM, 100 µM + 300 µM, and 300 µM + 1000 µM). The median effect dose (MD), in this case, the IC_50_ value, was taken as a measure of the drug’s potency. In particular, the values (reported in Table 2) were obtained by CompuSyn software v 1.0 (Combosyn, Inc, Paramus, NJ, USA) analysis, as the sum of the concentrations of the individual drugs that determine an IC50 when combined. For every CMT sample, the MD value of the combination in CMT*x* treatment is *y* micromolar, as 1/3 of this value corresponds to enrofloxacin, while 2/3 correspond to carboplatin. Table 2 reports MD values for single treatments and their combinations.

The results of cell viability in every condition are reported in Supplementary Online Material S1.

**Figure 5:**
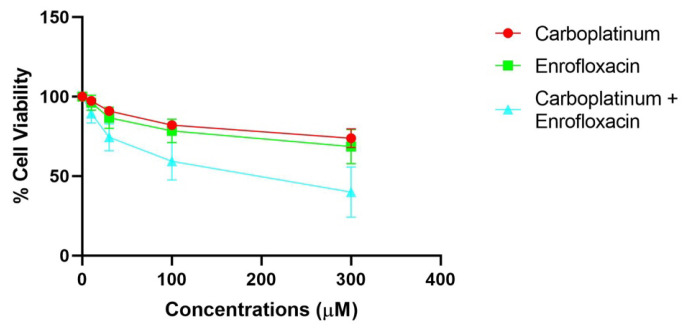
Survival data of four combination index plots obtained from MTT assays in some CMTs treated with single drugs and their combination, analyzed using CompuSyn software.

To assess the possible synergistic effect of the two drugs, we processed the MTT data using CompuSyn software v 1.0 (Combosyn, Inc, Paramus, NJ, USA) [58,59,60,61]. The results showed several synergistic points (C.I. < 1). Among those, three points showed additive effects (C.I. = 1)—CMT2, CMT3, and CMT23—and two points showed antagonistic effects (C.I. > 1)—CMT2 and CMT3. The median survival curve of all the treated samples is reported in Figure 5. Altogether, our results confirm the strongly increased sensitivity of the cells to the combo treatment with respect to the monotherapies.

Next, we used the Chou–Talalay formula [59,60,61] to calculate the Combination Index (CI) in order to assess if the two drugs act synergistically (CI < 1), additively (CI = 1), or antagonistically (CI > 1). With the exception of the minimum dosages in six tumors, the results showed that CI values were always <1 in all the combinations used, demonstrating the synergistic action of the two drugs (Supplementary Material S2). Therefore, enrofloxacin enables a significant reduction in the carboplatin dose while preserving its antiproliferative efficacy.

**Figure 6:**
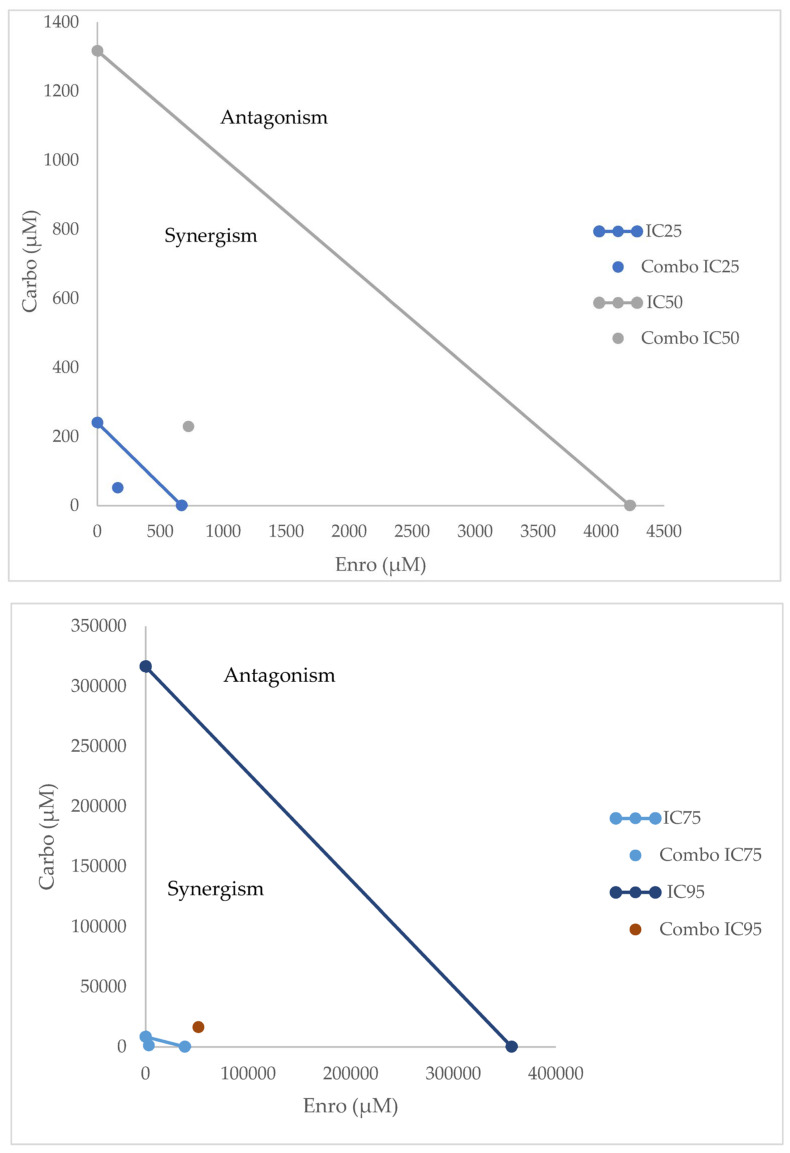
Isobolograms representing the mean drug concentrations corresponding to the IC25, IC50, IC75, and IC95 values of all samples. The y-axis indicates carboplatin concentrations, whereas the x-axis shows enrofloxacin concentrations. The mean concentrations normalized to the DRI are represented in the graphs as individual points.

Next, in order to evaluate if, thanks to the co-administration of enrofloxacin, it is possible to reduce the dosage of carboplatin while maintaining its efficacy, we determined the Dose Reduction Index (DRI) that estimates the extent to which carboplatin and enrofloxacin concentrations could be reduced while maintaining the same efficacy. Our results suggest that a lower concentration of carboplatin can achieve comparable therapeutic effects when used in combination with enrofloxacin, thereby potentially reducing chemotherapy-associated toxicity. We report the results obtained for a 95%, 75%, 50%, and 25% viability fraction, representing the IC25, IC50, IC75, and IC95 values of the treatments to provide a broader understanding of the dose–response relationship of the drug combination (Supplementary Material S3 and S4). The isobolograms of each sample obtained with CompuSyn analyses are reported in Supplementary Material S5. The median isobolograms of all four IC values studied are reported in Figure 6, summarizing the minimum effective concentrations achievable through the combination of the two drugs, followed by the median reduction in concentration values observed in the samples. The fraction affected indicates the proportion of inhibition or biological effect observed at a given dose of a drug or combination of drugs.

## 4. Discussion

CMTs are the most diagnosed malignancies in sexually intact female dogs [8]. According with the One Health approach, considering the shared environment and disease pathways, the study of animal diseases is desirable as the knowledge it provides is often transferable to human health and vice versa [36]. In this view, several aspects of mammary tumors (histology, diagnosis, mutations, and treatments) are shared between dogs and humans [11,12,13]. Therefore, CMTs represent a spontaneous model of HBC, and dogs can be considered a fruitful translational animal model for this human malignancy [23,24,26]. In dogs, the gold standard treatment for CMTs is surgery, although this approach is not definitive for all subtypes of CMT cases. Indeed, some dogs experience a tumor relapse, metastasis, and a reduction in survival [62,63]. In these cases, dogs can be treated pharmacologically with platinum-derived compounds (mainly carboplatin) that exert significant side effects including myelosuppression (neutropenia and thrombocytopenia), gastrointestinal dysfunctions and, less frequently, nephrotoxicity and ototoxicity. Moreover, residues of antineoplastic drugs in the urine, feces, and saliva of the treated dogs may represent exposure risks to veterinary personnel, owners and other animal care-takers [64]. In this context, lowering the carboplatin dose may also reduce its urinary excretion, thereby minimizing environmental risks and safety concerns. These adverse effects can limit tolerability and clinical use of platinum-based chemotherapy in veterinary oncology, underscoring the need for improved therapeutic strategies with better safety profiles and targeted efficacy.

In this context, our study is aimed to identify a treatment that could increase the outcome and the survival both in dogs and in humans affected by breast cancer.

Our previous studies in mouse models and in a genetically modified human cell model highlighted the crucial role of the MCM2 protein in tumor growth [43]. At the same time, other studies on breast cancer corroborated this role [42,43,45]. In addition, other studies reported an increased susceptibility of tumor cells to the platinum compound using fluoroquinolones (inhibitors of MCM2 function) in the treatment of HBC [49,51]. Together, all these findings suggest the repositioning of fluoroquinolones from antibiotics to anticancer agents although the correlation between MCM protein expression and the tumor grade should be investigated in detail as a possible prognostic tool [52,53,54,56]. This information led us to hypothesize the possible inhibition of MCM2 function by fluoroquinolones as a potential anticancer co-therapy. Enrofloxacin is a fluoroquinolone commonly used in veterinary practice, with ciprofloxacin being its counterpart in humans. In this work we examined the possibility of using enrofloxacin as an anticancer agent in primary CMT explants in co-administration with carboplatin, testing their possible synergy in order to possibly diminish the side effects of platinum while maintaining the same level of efficacy.

We found that treating CMTs with a combination of enrofloxacin and carboplatin results in an increased reduction in cell proliferation, reduced cell viability with respect to carboplatin as a single agent and enhanced anticancer drug potency without a significant increase in toxic effects. Our results showed that the addition of a relatively non-toxic molecule such as a fluoroquinolone allows for a drastic reduction in the therapeutic dosage of an extremely toxic molecule such as carboplatin, thus decreasing its side effects.

In three CMTs (TM23, 31 and 53), the response to treatments, both individually and in combination, resulted in a comparable effect. Interestingly, these were benign tumors (see Table 1) and, as such, more susceptible to the antiproliferative effects of platinum, as they lack some molecular mechanisms responsible for resistance to chemotherapeutics, which instead are usually expressed in cells with acquired malignancy. Similarly, the greater response of enrofloxacin in benign tumors could be determined by reduced resistance mechanisms in these tumors compared to malignant tumors. Furthermore, malignant tumors have a higher propensity to alter their genome because of increased genomic instability, thereby potentially altering drug targets and becoming resistant to treatments. In benign tumors, these events are less frequent and may contribute to a greater sensitivity to the drug.

We also evaluated the possibility of maintaining the normal therapeutic effects of platinum while reducing the administered doses by its co-administration with enrofloxacin. The results showed that the combination of enrofloxacin with carboplatin allows for a reduction in carboplatin doses without compromising cytotoxic efficacy, thus making it administrable even in cases of low tolerance of toxic effects. At the same time, it is also possible to lower the doses of fluoroquinolones, thereby mitigating potential toxic effects associated with their use while maintaining comparable therapeutic activity. Further in-depth studies will clarify whether the same therapeutic benefit is maintained at lower dosages or whether, alternatively, the benefit is less but still significant.

These findings open the possibility of administering carboplatin in animals with renal or bone marrow impairment by reducing the dose and, consequently, the common side effects often observed in dogs. Moreover, lowering the dosages could shorten the duration and decrease the costs of intravenous treatment, improving owner compliance and overall acceptance of the therapy. These aspects currently represent major limitations to the practical use of carboplatin in veterinary oncology. Additionally, lowering the carboplatin dose may also reduce its urinary excretion, thereby minimizing environmental risks and safety concerns for pet owners. Considering that CMTs are molecularly more like HBCs, our results provide a way to investigate the possibility of a new, alternative therapeutic approach for these tumors.

However, important genetic and phenotypic differences clearly distinguish between CMTs and HBC. In dogs, tumors are more frequently identified as so-called “complex” carcinomas (proliferation of myoepithelial and mesenchymal components), which are rare in humans [65]. Triple-negative subtypes in dogs have emerged as a valuable comparative model for TNBC, sharing several clinicopathological and molecular features while retaining species-specific differences. At the gene level, mutations in some key genes show different frequencies in the two species (i.e., MKI67, Ki-67, KRAS, TP53, TTN, and CDH1 [31,32].

## 5. Conclusions

In conclusion, although this study supports the use of the dog as animal models for human cancer studies, in line with the One Health frame, further investigations are needed before the study can be translated into human clinical practice. From this point of view, the use of drugs already approved for treatments in dogs can speed up the applicability of the study in the veterinary clinic.

Finally, a more detailed investigation of the effects of the combination therapy could be obtained with future mechanistic studies.

## Figures and Tables

**Table 1:** CL Davis Foundation Criteria of Canine Mammary Tumors were adopted to classify the samples analyzed in histology. Different grades of malignancy were determined. Benign tumors are also reported in the list.

Samples	Histology
CMT 1	Grade I simple carcinoma and tubulo-papillary carcinoma
CMT 2	Grade I complex carcinoma
CMT 3	Grade I complex carcinoma and papillary carcinoma
CMT 4	Benign mixed tumor and lobular hyperplasia
CMT 23	Benign mixed tumor
CMT 26	Grade II adenosquamous carcinoma
CMT 28	Grade II complex carcinoma
CMT 30	Complex adenoma
CMT 31	Benign mixed tumor associated with hyperplasia
CMT 34	Grade I simple tubular adenocarcinoma
CMT 46	Grade I complex adenocarcinoma with lobular hyperplasia
CMT 47	Grade I intraductal papillary carcinoma and duct ectasia
CMT 48	Grade II simple tubular carcinoma
CMT 50	Grade II adenosquamous carcinoma and grade II tubulo-papillary carcinoma
CMT 51	Grade I complex carcinoma
CMT 52	Benign adenoma and lobular hyperplasia
CMT 53	Benign mixed tumor

**Table 2:** The MD value of the combination considers an approximately 1:3 ratio between the two drugs (carboplatin 1: enrofloxacin 3).

Samples	Carbo IC50 (µM)	Enro IC50 (µM)	Carbo-Enro IC50 (µM)
CMT1	1526.470	1577.450	283.911
CMT2	467.199	771.439	450.663
CMT3	1589.730	2853.910	412.451
CMT4	3577.980	10,883.800	2941.880
CMT23	427.221	952.923	1851.920
CMT26	1870.760	17,013.100	575.250
CMT30	689.009	1536.950	310.133
CMT47	893.288	2844.470	1202.470
CMT50	1781.200	2756.880	1037.630
CMT51	347.323	1100.910	471.433

## Data Availability

The original contributions presented in this study are included in the article/Supplementary Material. Further inquiries can be directed to the corresponding author(s).

## Supplementary Materials

The following supporting information can be downloaded at: https://www.mdpi.com/article/10.3390/biology15080604/s1.
