# Supplementary material for: The Co-Administration of Fluoroquinolones Strongly Increases the Anticancer Efficacy of Carboplatin Treatment—Novel Insights for Breast Cancer Chemotherapy from the Canine Mammary Tumor Model
Source: Biology (Basel). 2026 Apr 11;15(8):604. doi: 10.3390/biology15080604 (PMC13113806; doi:10.3390/biology15080604)
Supplement: Supplementary file 1 [file biology-15-00604-s001.zip › Supplementary Material 1.pptx]

## Slide 1
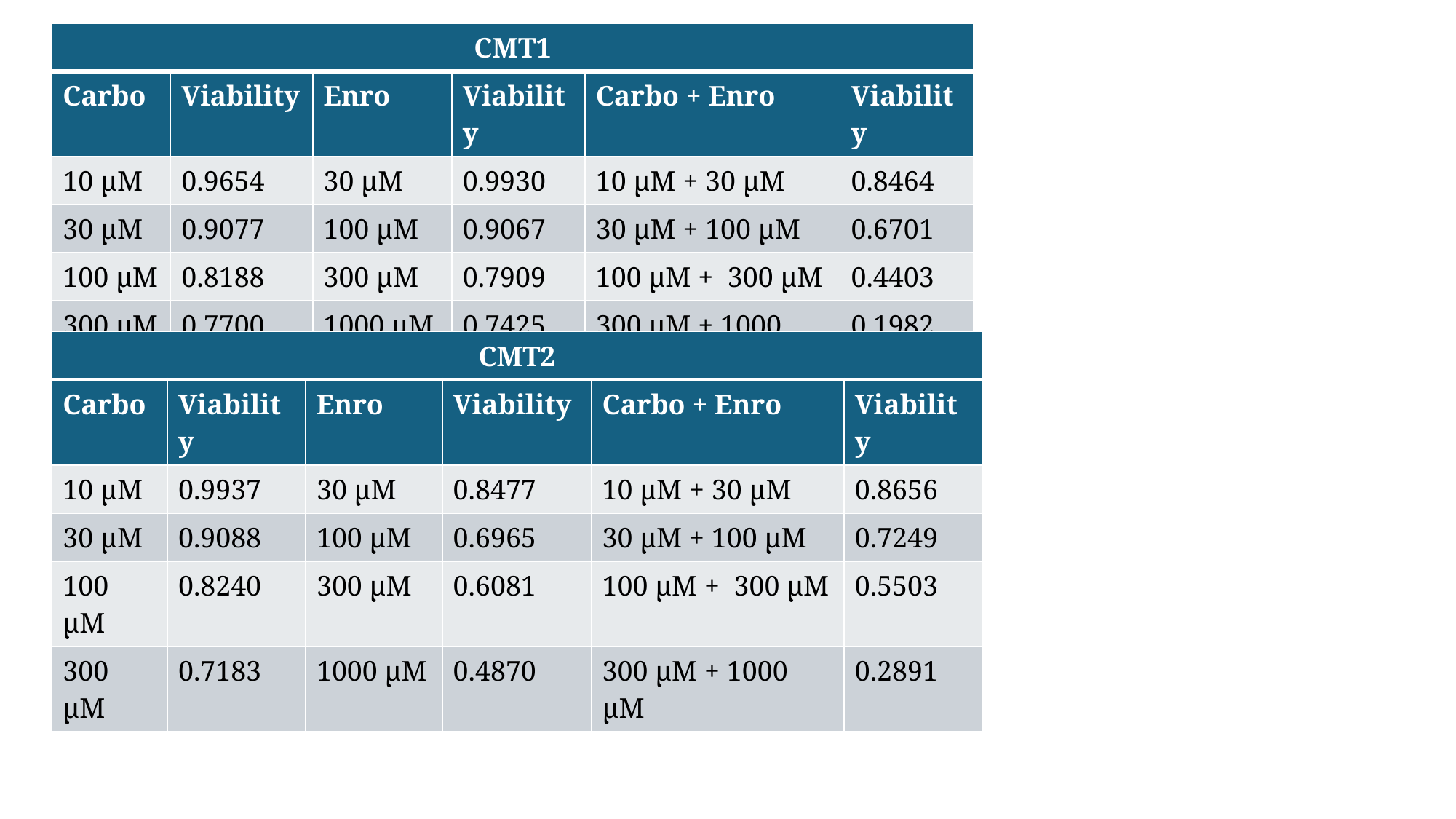

| CMT1 | | | | | |
| --- | --- | --- | --- | --- | --- |
| Carbo | Viability | Enro | Viability | Carbo + Enro | Viability |
| 10 µM | 0.9654 | 30 µM | 0.9930 | 10 µM + 30 µM | 0.8464 |
| 30 µM | 0.9077 | 100 µM | 0.9067 | 30 µM + 100 µM | 0.6701 |
| 100 µM | 0.8188 | 300 µM | 0.7909 | 100 µM + 300 µM | 0.4403 |
| 300 µM | 0.7700 | 1000 µM | 0.7425 | 300 µM + 1000 µM | 0.1982 |
| CMT2 | | | | | |
| --- | --- | --- | --- | --- | --- |
| Carbo | Viability | Enro | Viability | Carbo + Enro | Viability |
| 10 µM | 0.9937 | 30 µM | 0.8477 | 10 µM + 30 µM | 0.8656 |
| 30 µM | 0.9088 | 100 µM | 0.6965 | 30 µM + 100 µM | 0.7249 |
| 100 µM | 0.8240 | 300 µM | 0.6081 | 100 µM + 300 µM | 0.5503 |
| 300 µM | 0.7183 | 1000 µM | 0.4870 | 300 µM + 1000 µM | 0.2891 |

## Slide 2
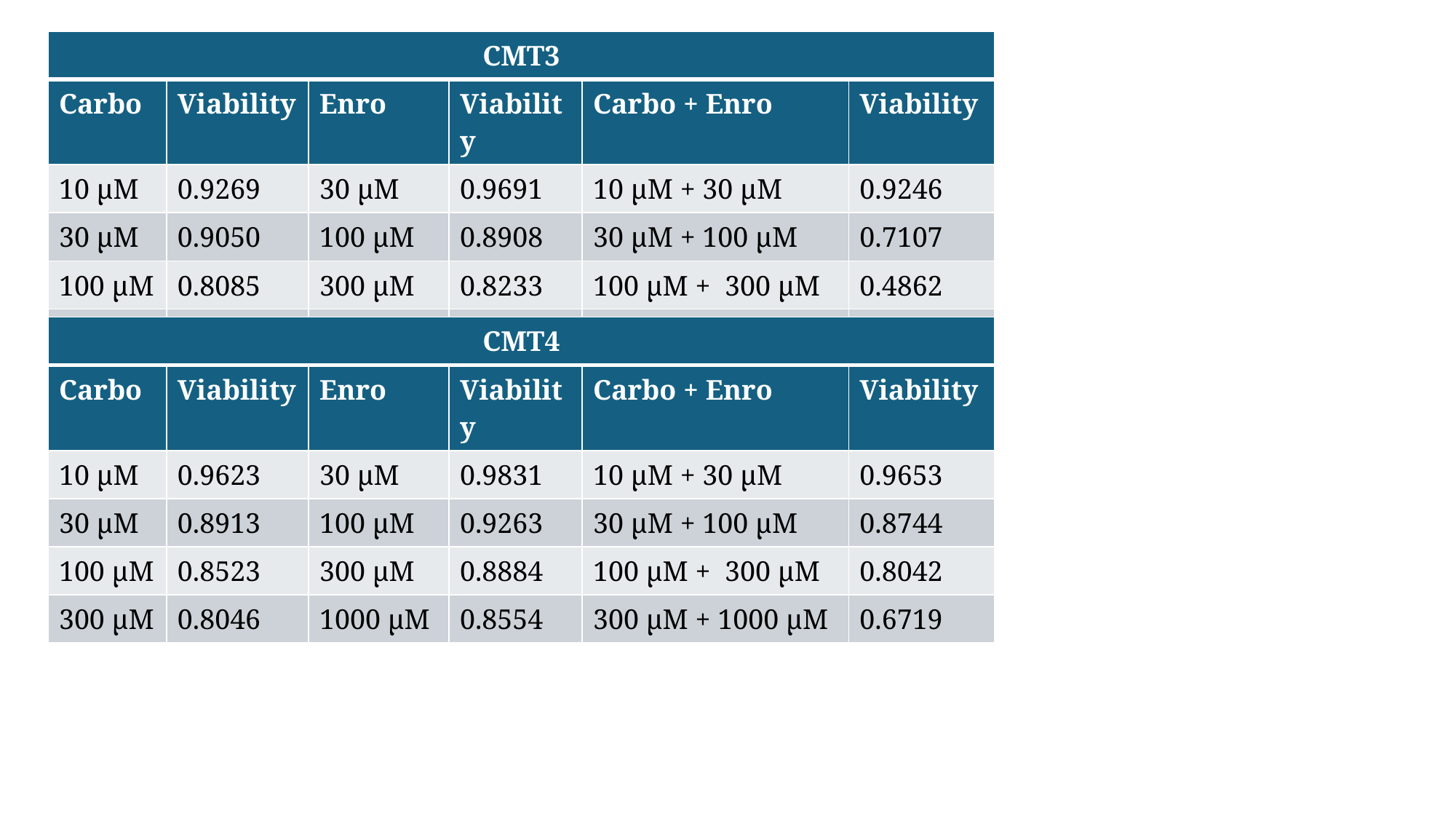

| CMT3 | | | | | |
| --- | --- | --- | --- | --- | --- |
| Carbo | Viability | Enro | Viability | Carbo + Enro | Viability |
| 10 µM | 0.9269 | 30 µM | 0.9691 | 10 µM + 30 µM | 0.9246 |
| 30 µM | 0.9050 | 100 µM | 0.8908 | 30 µM + 100 µM | 0.7107 |
| 100 µM | 0.8085 | 300 µM | 0.8233 | 100 µM + 300 µM | 0.4862 |
| 300 µM | 0.6949 | 1000 µM | 0.7076 | 300 µM + 1000 µM | 0.2692 |
| CMT4 | | | | | |
| --- | --- | --- | --- | --- | --- |
| Carbo | Viability | Enro | Viability | Carbo + Enro | Viability |
| 10 µM | 0.9623 | 30 µM | 0.9831 | 10 µM + 30 µM | 0.9653 |
| 30 µM | 0.8913 | 100 µM | 0.9263 | 30 µM + 100 µM | 0.8744 |
| 100 µM | 0.8523 | 300 µM | 0.8884 | 100 µM + 300 µM | 0.8042 |
| 300 µM | 0.8046 | 1000 µM | 0.8554 | 300 µM + 1000 µM | 0.6719 |

## Slide 3
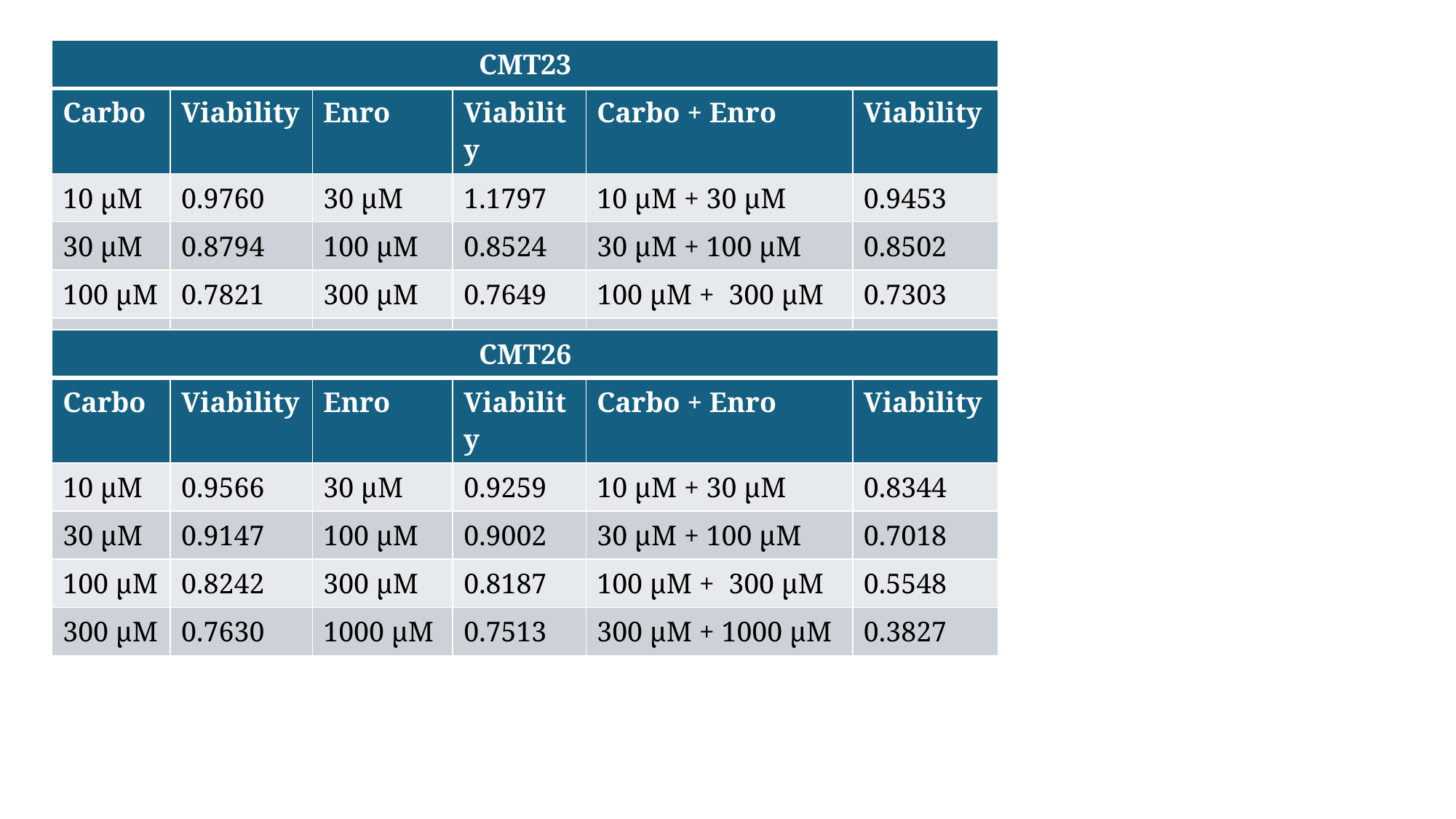

| CMT23 | | | | | |
| --- | --- | --- | --- | --- | --- |
| Carbo | Viability | Enro | Viability | Carbo + Enro | Viability |
| 10 µM | 0.9760 | 30 µM | 1.1797 | 10 µM + 30 µM | 0.9453 |
| 30 µM | 0.8794 | 100 µM | 0.8524 | 30 µM + 100 µM | 0.8502 |
| 100 µM | 0.7821 | 300 µM | 0.7649 | 100 µM + 300 µM | 0.7303 |
| 300 µM | 0.6187 | 1000 µM | 0.5830 | 300 µM + 1000 µM | 0.5894 |
| CMT26 | | | | | |
| --- | --- | --- | --- | --- | --- |
| Carbo | Viability | Enro | Viability | Carbo + Enro | Viability |
| 10 µM | 0.9566 | 30 µM | 0.9259 | 10 µM + 30 µM | 0.8344 |
| 30 µM | 0.9147 | 100 µM | 0.9002 | 30 µM + 100 µM | 0.7018 |
| 100 µM | 0.8242 | 300 µM | 0.8187 | 100 µM + 300 µM | 0.5548 |
| 300 µM | 0.7630 | 1000 µM | 0.7513 | 300 µM + 1000 µM | 0.3827 |

## Slide 4
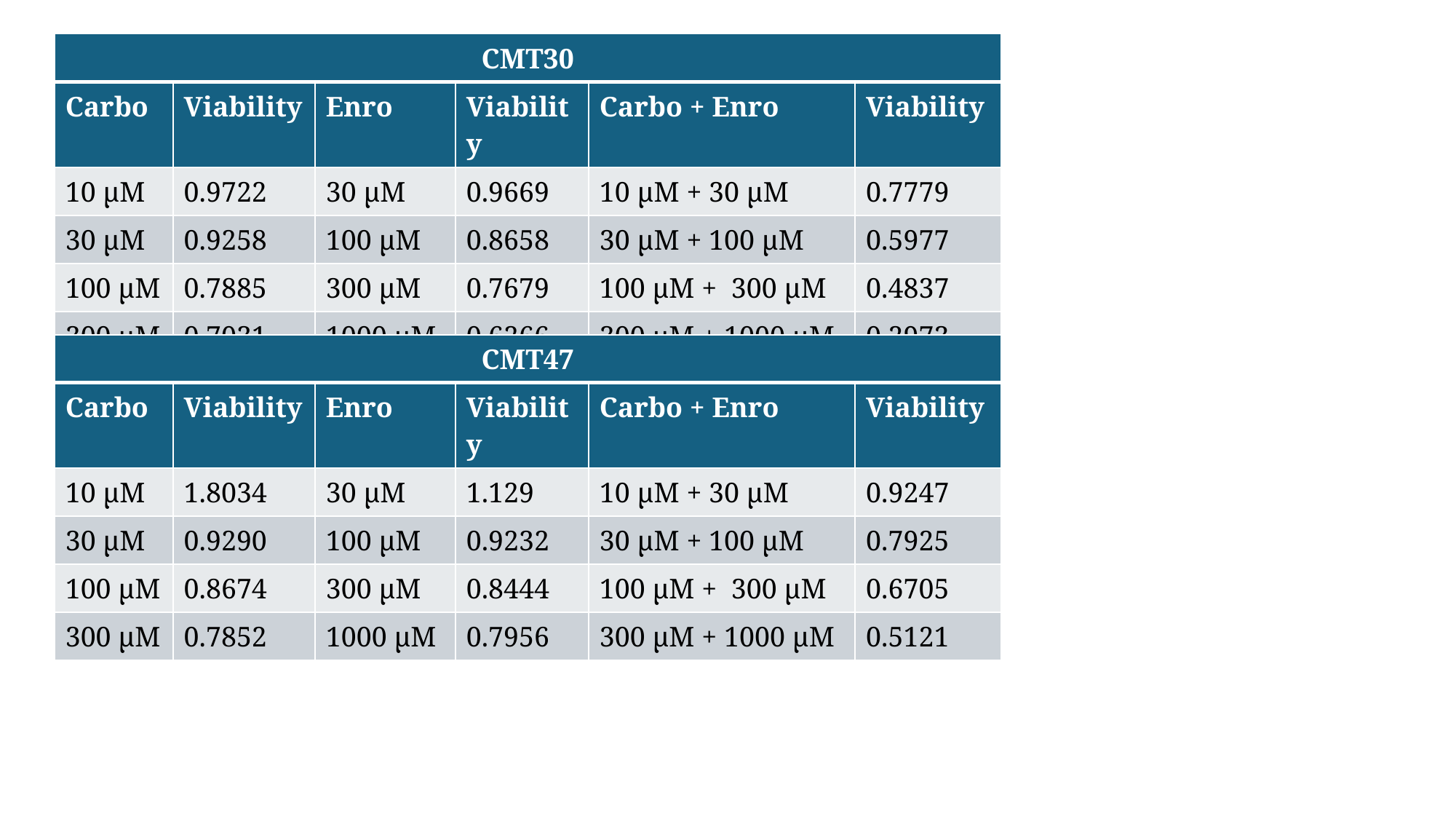

| CMT30 | | | | | |
| --- | --- | --- | --- | --- | --- |
| Carbo | Viability | Enro | Viability | Carbo + Enro | Viability |
| 10 µM | 0.9722 | 30 µM | 0.9669 | 10 µM + 30 µM | 0.7779 |
| 30 µM | 0.9258 | 100 µM | 0.8658 | 30 µM + 100 µM | 0.5977 |
| 100 µM | 0.7885 | 300 µM | 0.7679 | 100 µM + 300 µM | 0.4837 |
| 300 µM | 0.7031 | 1000 µM | 0.6266 | 300 µM + 1000 µM | 0.2973 |
| CMT47 | | | | | |
| --- | --- | --- | --- | --- | --- |
| Carbo | Viability | Enro | Viability | Carbo + Enro | Viability |
| 10 µM | 1.8034 | 30 µM | 1.129 | 10 µM + 30 µM | 0.9247 |
| 30 µM | 0.9290 | 100 µM | 0.9232 | 30 µM + 100 µM | 0.7925 |
| 100 µM | 0.8674 | 300 µM | 0.8444 | 100 µM + 300 µM | 0.6705 |
| 300 µM | 0.7852 | 1000 µM | 0.7956 | 300 µM + 1000 µM | 0.5121 |

## Slide 5
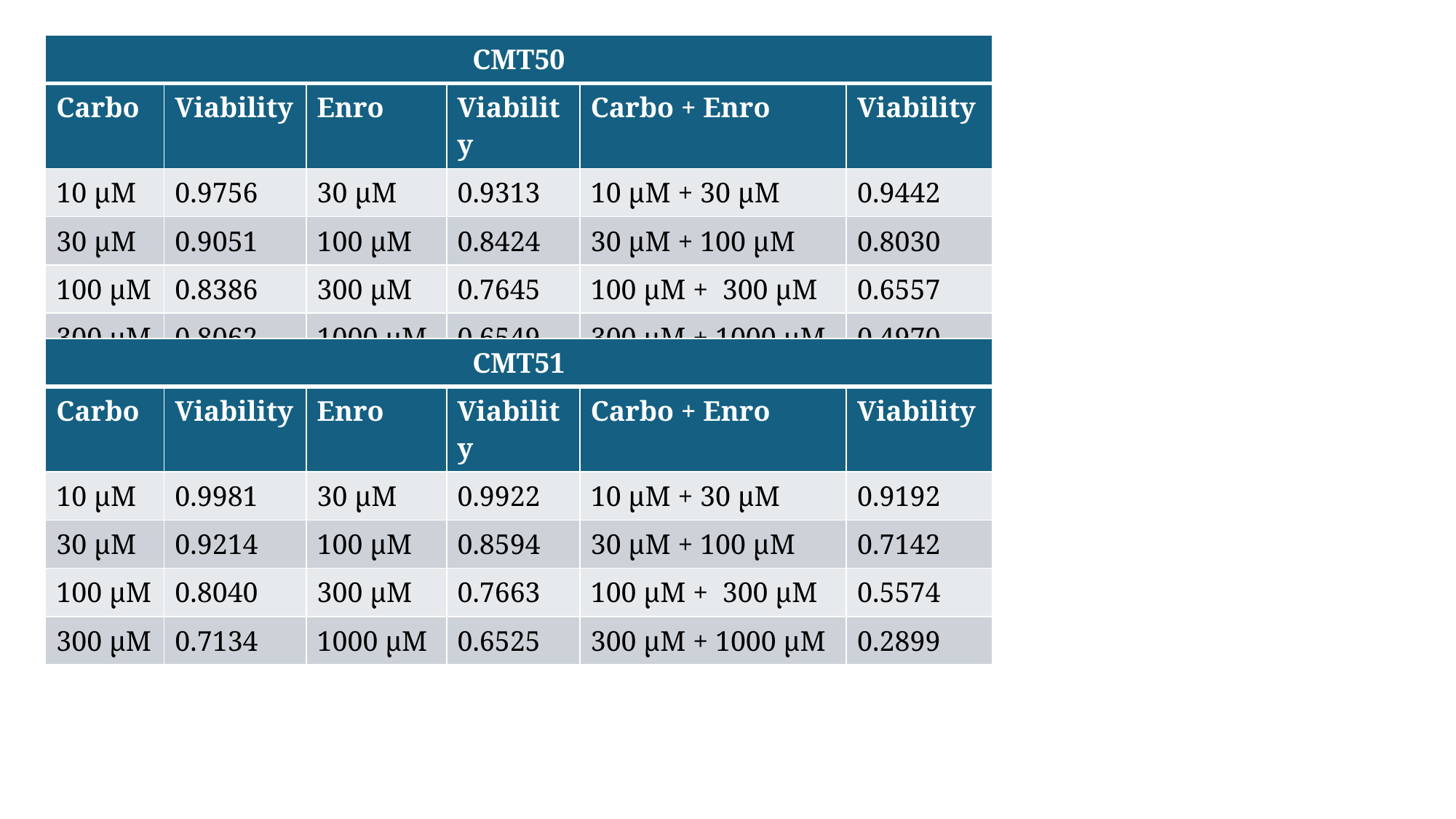

| CMT50 | | | | | |
| --- | --- | --- | --- | --- | --- |
| Carbo | Viability | Enro | Viability | Carbo + Enro | Viability |
| 10 µM | 0.9756 | 30 µM | 0.9313 | 10 µM + 30 µM | 0.9442 |
| 30 µM | 0.9051 | 100 µM | 0.8424 | 30 µM + 100 µM | 0.8030 |
| 100 µM | 0.8386 | 300 µM | 0.7645 | 100 µM + 300 µM | 0.6557 |
| 300 µM | 0.8062 | 1000 µM | 0.6549 | 300 µM + 1000 µM | 0.4970 |
| CMT51 | | | | | |
| --- | --- | --- | --- | --- | --- |
| Carbo | Viability | Enro | Viability | Carbo + Enro | Viability |
| 10 µM | 0.9981 | 30 µM | 0.9922 | 10 µM + 30 µM | 0.9192 |
| 30 µM | 0.9214 | 100 µM | 0.8594 | 30 µM + 100 µM | 0.7142 |
| 100 µM | 0.8040 | 300 µM | 0.7663 | 100 µM + 300 µM | 0.5574 |
| 300 µM | 0.7134 | 1000 µM | 0.6525 | 300 µM + 1000 µM | 0.2899 |
