# Supplementary material for: The Co-Administration of Fluoroquinolones Strongly Increases the Anticancer Efficacy of Carboplatin Treatment—Novel Insights for Breast Cancer Chemotherapy from the Canine Mammary Tumor Model
Source: Biology (Basel). 2026 Apr 11;15(8):604. doi: 10.3390/biology15080604 (PMC13113806; doi:10.3390/biology15080604)
Supplement: Supplementary file 1 [file biology-15-00604-s001.zip › Supplementary Material 2.pdf]

Combo Concentrations (Carbo + Enro)	CMT1 F.A.	CMT1 C.I.	CMT2 F.A.	CMT2 C.I.	CMT3 F.A.	CMT3 C.I.
**10 μM + 30 μM**	**0,846**	**0,188**	**0,865**	**1,852**	**0,924**	**1,085**
**30 μM + 100 μM**	**0,670**	**0,183**	**0,725**	**1,076**	**0,710**	**0,231**
**100 μM + 300 μM**	**0,440**	**0,197**	**0,550**	**0,840**	**0,486**	**0,152**
**300 μM + 1000 μM**	**0,198**	**0,195**	**0,289**	**0,510**	**0,269**	**0,114**
**Combo Concentrations (Carbo + Enro)**	**CMT4 F.A.**	**CMT4 C.I.**	**CMT23 F.A.**	**CMT23 C.I.**	**CMT26 F.A.**	**CMT26 C.I.**
**10 μM + 30 μM**	**0,965**	**2,387**	**0,945**	**0,897**	**0,834**	**0,180**
**30 μM + 100 μM**	**0,874**	**0,591**	**0,850**	**0,962**	**0,701**	**0,121**
**100 μM + 300 μM**	**0,804**	**0,692**	**0,730**	**1,430**	**0,554**	**0,105**
**300 μM + 1000 μM**	**0,671**	**0,639**	**0,589**	**2,506**	**0,382**	**0,090**
**Combo Concentrations (Carbo + Enro)**	**CMT30 F.A.**	**CMT30 C.I.**	**CMT47 F.A.**	**CMT47 C.I.**	**CMT50 F.A.**	**CMT50 C.I.**
**10 μM + 30 μM**	**0,777**	**0,160**	**0,924**	**0,332**	**0,944**	**2,292**
**30 μM + 100 μM**	**0,597**	**0,179**	**0,792**	**0,301**	**0,803**	**0,612**
**100 μM + 300 μM**	**0,483**	**0,310**	**0,670**	**0,465**	**0,655**	**0,500**
**300 μM + 1000 μM**	**0,297**	**0,375**	**0,512**	**0,734**	**0,497**	**0,522**
**Combo Concentrations (Carbo + Enro)**	**CMT51 F.A.**	**CMT51 C.I.**				
**10 μM + 30 μM**	**0,9191**	**0,370**				
**30 μM + 100 μM**	**0,714**	**0,365**				
**100 μM + 300 μM**	**0,557**	**0,660**				
**300 μM + 1000 μM**	**0,289**	**0,904**				

Supplementary Material 2 - The table reports the values of the fraction affected (F.A.) and the combination index (C.I.) of the samples.
