# Supplementary material for: The Co-Administration of Fluoroquinolones Strongly Increases the Anticancer Efficacy of Carboplatin Treatment—Novel Insights for Breast Cancer Chemotherapy from the Canine Mammary Tumor Model
Source: Biology (Basel). 2026 Apr 11;15(8):604. doi: 10.3390/biology15080604 (PMC13113806; doi:10.3390/biology15080604)
Supplement: Supplementary file 1 [file biology-15-00604-s001.zip › Supplementary Material 3.pdf]

	F.A. 0.25		F.A. 0.5		F.A. 0.75		F.A. 0.95	
Samples	DRI Carbo	DRI Enro	DRI Carbo	DRI Enro	DRI Carbo	DRI Enro	DRI Carbo	DRI Enro
CMT 1	37,55	5,81	22,39	7,31	13,35	9,19	5,6	13,5
CMT 2	2,76	5,18	4,31	2,25	6,74	0,97	14,25	0,24
CMT 3	43,19	14,1	16,05	9,1	5,96	5,87	1,13	2,81
CMT 4	9,59	6,07	5,06	4,86	2,67	3,89	0,91	2,68
CMT 23	0,68	0,37	0,96	0,67	1,34	1,22	2,37	3,34
CMT 26	14,39	93,27	13,54	38,91	12,74	16,23	11,49	3,73
CMT 30	5,5	4	9,25	6,52	15,54	10,61	37,17	24,04
CMT 47	2,07	2,14	3,09	3,11	4,61	4,51	9,02	8,43
CMT 50	9,92	6,53	7,14	3,49	5,14	1,87	2,96	0,65
CMT 51	1,94	2,43	3,06	3,07	4,84	3,87	10,41	5,71
Medium	12,75	13,99	8,48	7,92	7,29	5,82	9,53	6,53

Supplementary Material 3 – The table included individual DRI values, and corresponding means, at different FA levels (0.25, 0.50, 0.75, 0.95).
