# Supplementary material for: The Co-Administration of Fluoroquinolones Strongly Increases the Anticancer Efficacy of Carboplatin Treatment—Novel Insights for Breast Cancer Chemotherapy from the Canine Mammary Tumor Model
Source: Biology (Basel). 2026 Apr 11;15(8):604. doi: 10.3390/biology15080604 (PMC13113806; doi:10.3390/biology15080604)
Supplement: Supplementary file 1 [file biology-15-00604-s001.zip › Supplementary Material 4.pdf]

Samples	Cell Viability	Conc. Carbo (µM)	Conc. Enro (µM)	Carbo/DRI (µM)	Enro/DRI (µM)
CMT1	IC25	263,888	574,934	19,76	62,539
CMT2	IC25	179,366	82,388	26,601	84,192
CMT3	IC25	195,215	608,481	32,717	103,55
CMT4	IC25	413,576	1907	154,556	489,171
CMT23	IC25	127,273	367,483	94,504	299,108
CMT26	IC25	280,658	1132,2	22,028	69,719
CMT30	IC25	179,033	386,761	11,5157	36,447
CMT47	IC25	272,748	844,98	59,1135	187,094
CMT50	IC25	326,412	375,635	63,408	200,686
CMT51	IC25	166,605	421,908	34,4161	108,927
	**Mean**	240,4774	670,177	51,86193	164,1433
**Samples**	**Cell Viability**	**Conc. Carbo (µM)**	**Conc. Enro (µM)**	**Carbo/DRI (µM)**	**Enro/DRI (µM)**
CMT1	IC50	1526,47	1577,45	68,165	215,745
CMT2	IC50	467,199	771,439	108,202	342,461
CMT3	IC50	1589,73	2853,91	99,027	313,423
CMT4	IC50	3577,98	10883,8	706,333	2235,54

CMT23	IC50	427,221	952,923	444,64	1407,28
CMT26	IC50	1870,76	17013,1	138,115	437,135
CMT30	IC50	689,009	1536,95	74,461	235,672
CMT47	IC50	893,288	2844,47	288,131	913,765
CMT50	IC50	1781,2	2756,88	249,131	788,5
CMT51	IC50	347,323	1100,91	113,189	358,244
	**Mean**	1317,018	4229,1832	228,9972	724,7765

**Samples**	**Cell Viability**	**Conc. Carbo (µM)**	**Conc. Enro (µM)**	**Carbo/DRI (µM)**	**Enro/DRI (µM)**
CMT1	**IC75**	8829,94	4328,07	235,154	744,262
CMT2	**IC75**	1216,93	7223,35	440,124	1392,99
CMT3	**IC75**	12945,9	13385,5	299,737	948,668
CMT4	**IC75**	30954,3	62116,7	3227,99	10216,6
CMT23	**IC75**	1434,06	2471,03	2092	6621,19
CMT26	**IC75**	12469,8	255647	865,966	2740,78
CMT30	**IC75**	2651,65	6107,65	481,478	1523,88
CMT47	**IC75**	2925,64	9575,37	1410,05	4462,81

CMT50	**IC75**	9719,84	20233,4	978,84	3098,03
CMT51	**IC75**	724,065	2872,67	372,263	1178,21
	**Mean**	8387,2125	38396,074	1040,3602	3292,742
**Samples**	**Cell Viability**	**Conc. Carbo (µM)**	**Conc. Enro (µM)**	**Carbo/DRI (µM)**	**Enro/DRI (µM)**
CMT1	**IC95**	168531	23592,1	1883,448	5960,611
CMT2	**IC95**	6078,62	309665	4650,818	14713,722
CMT3	**IC95**	438958	179613	1926,902	6098,913
CMT4	**IC95**	11611861	1159092	41480,221	131252,633
CMT23	**IC95**	10969,3	12250,5	28220,478	89419,708
CMT26	**IC95**	1161861	1159092	41466,897	131252,633
CMT30	**IC95**	25520,5	62035,8	11095,87	35088,122
CMT47	**IC95**	21472,5	73595,5	20257,07	64107,57
CMT50	**IC95**	168203	576085	9756,554	30872,72
CMT51	**IC95**	2487,81	14392	2752,002	8711,864
	**Mean**	316594,273	356941,29	16349,026	51749,336

Supplementary Material 4 – The table reports the IC25, IC50, IC75, and IC95 values for all samples in response to treatment with carboplatin and enrofloxacin, along with the mean value for each parameter. The last two columns show the drug concentration values normalized to the DRI for all treated samples, including the corresponding means.
