# Supplementary material for: The Co-Administration of Fluoroquinolones Strongly Increases the Anticancer Efficacy of Carboplatin Treatment—Novel Insights for Breast Cancer Chemotherapy from the Canine Mammary Tumor Model
Source: Biology (Basel). 2026 Apr 11;15(8):604. doi: 10.3390/biology15080604 (PMC13113806; doi:10.3390/biology15080604)
Supplement: Supplementary file 1 [file biology-15-00604-s001.zip › Supplementary Material 5.pptx]

## Slide 1
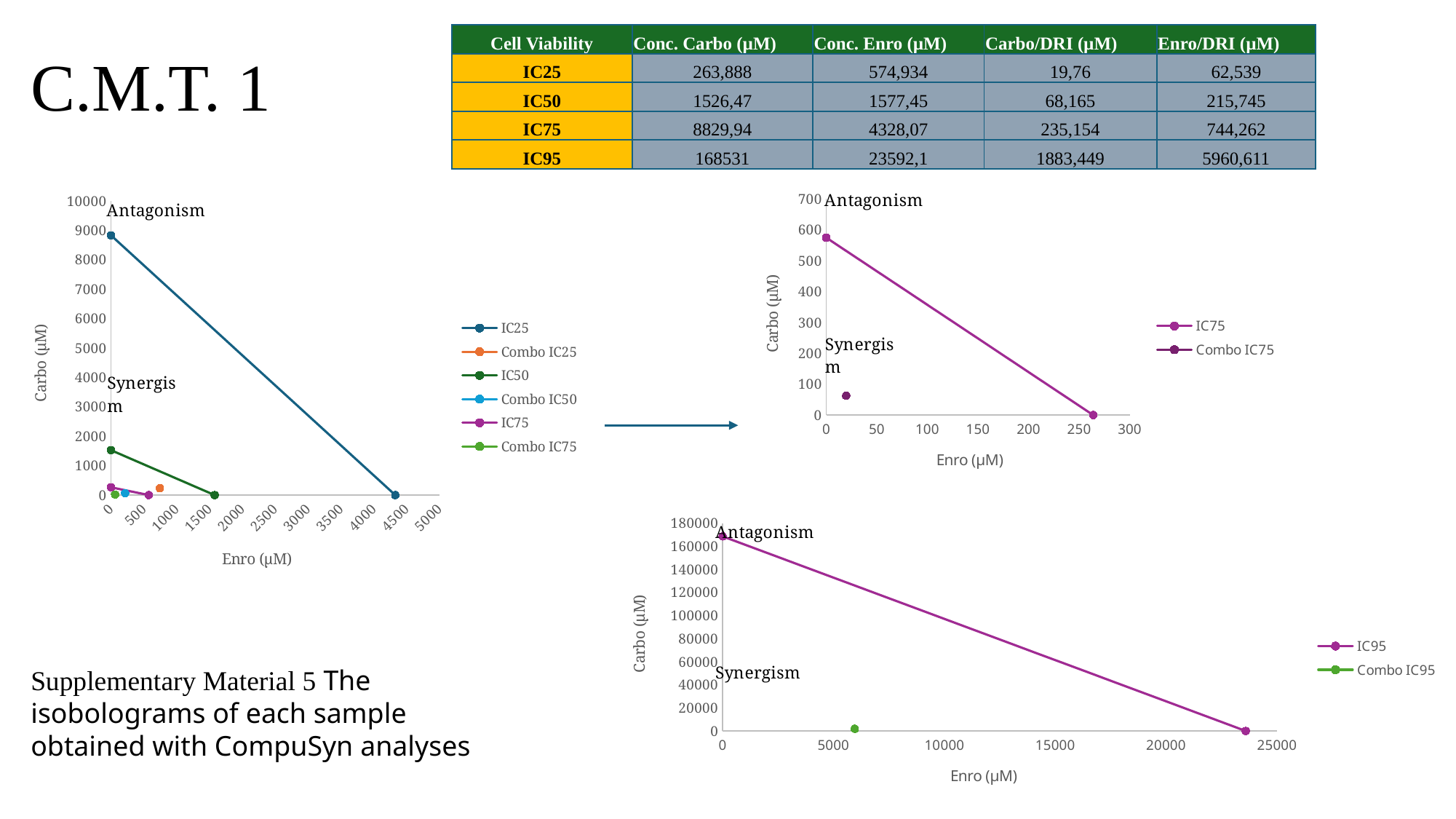

# C.M.T. 1
| Cell Viability | Conc. Carbo (µM) | Conc. Enro (µM) | Carbo/DRI (µM) | Enro/DRI (µM) |
| --- | --- | --- | --- | --- |
| IC25 | 263,888 | 574,934 | 19,76 | 62,539 |
| IC50 | 1526,47 | 1577,45 | 68,165 | 215,745 |
| IC75 | 8829,94 | 4328,07 | 235,154 | 744,262 |
| IC95 | 168531 | 23592,1 | 1883,449 | 5960,611 |
### Chart
| Category | | | | | | |
|---|---|---|---|---|---|---|
### Chart
| Category | | |
|---|---|---|
### Chart
| Category | | |
|---|---|---|Supplementary Material 5 The isobolograms of each sample obtained with CompuSyn analyses

## Slide 2
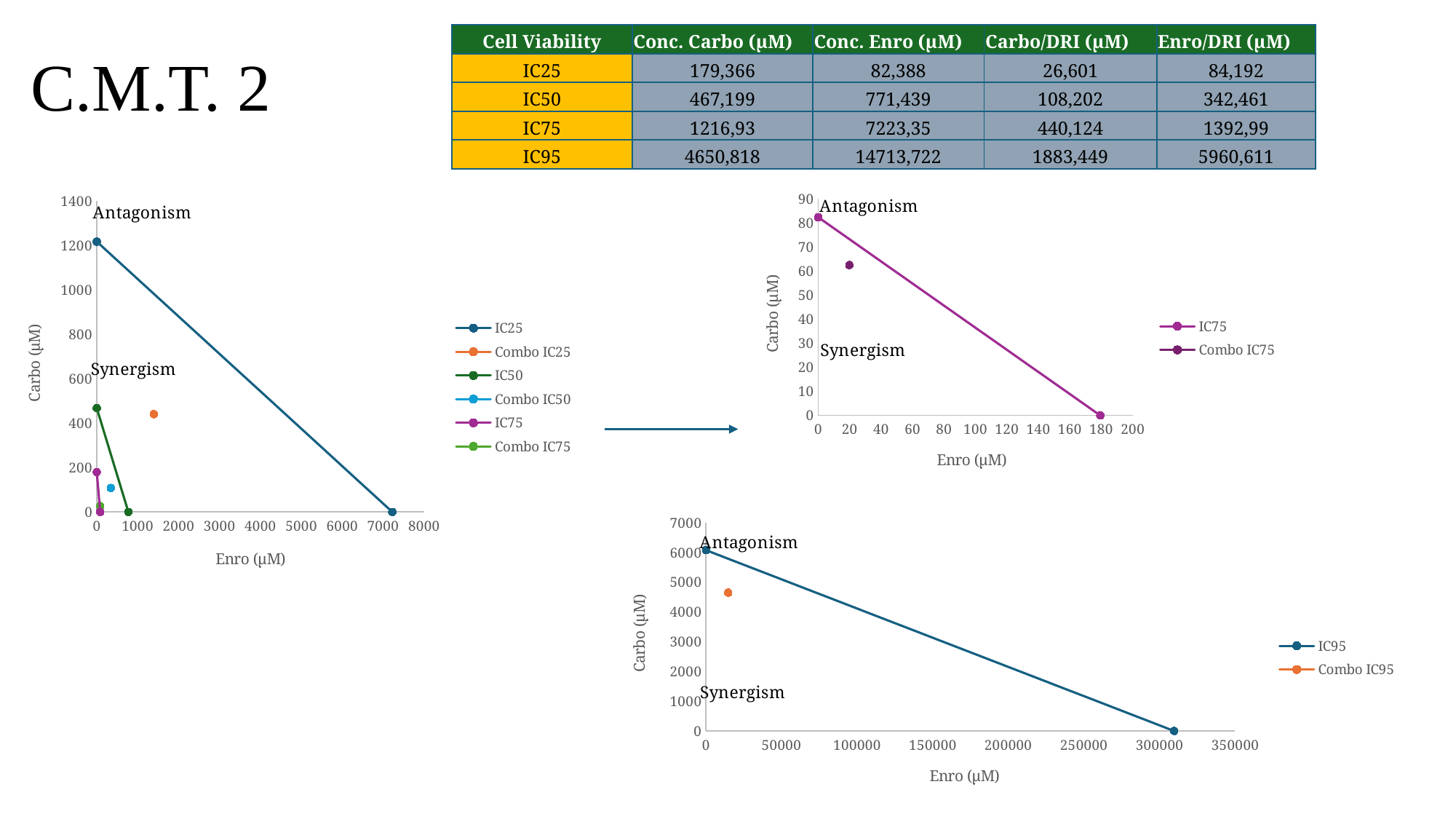

# C.M.T. 2
| Cell Viability | Conc. Carbo (µM) | Conc. Enro (µM) | Carbo/DRI (µM) | Enro/DRI (µM) |
| --- | --- | --- | --- | --- |
| IC25 | 179,366 | 82,388 | 26,601 | 84,192 |
| IC50 | 467,199 | 771,439 | 108,202 | 342,461 |
| IC75 | 1216,93 | 7223,35 | 440,124 | 1392,99 |
| IC95 | 4650,818 | 14713,722 | 1883,449 | 5960,611 |
### Chart
| Category | | | | | | |
|---|---|---|---|---|---|---|
### Chart
| Category | | |
|---|---|---|
### Chart
| Category | | |
|---|---|---|

## Slide 3
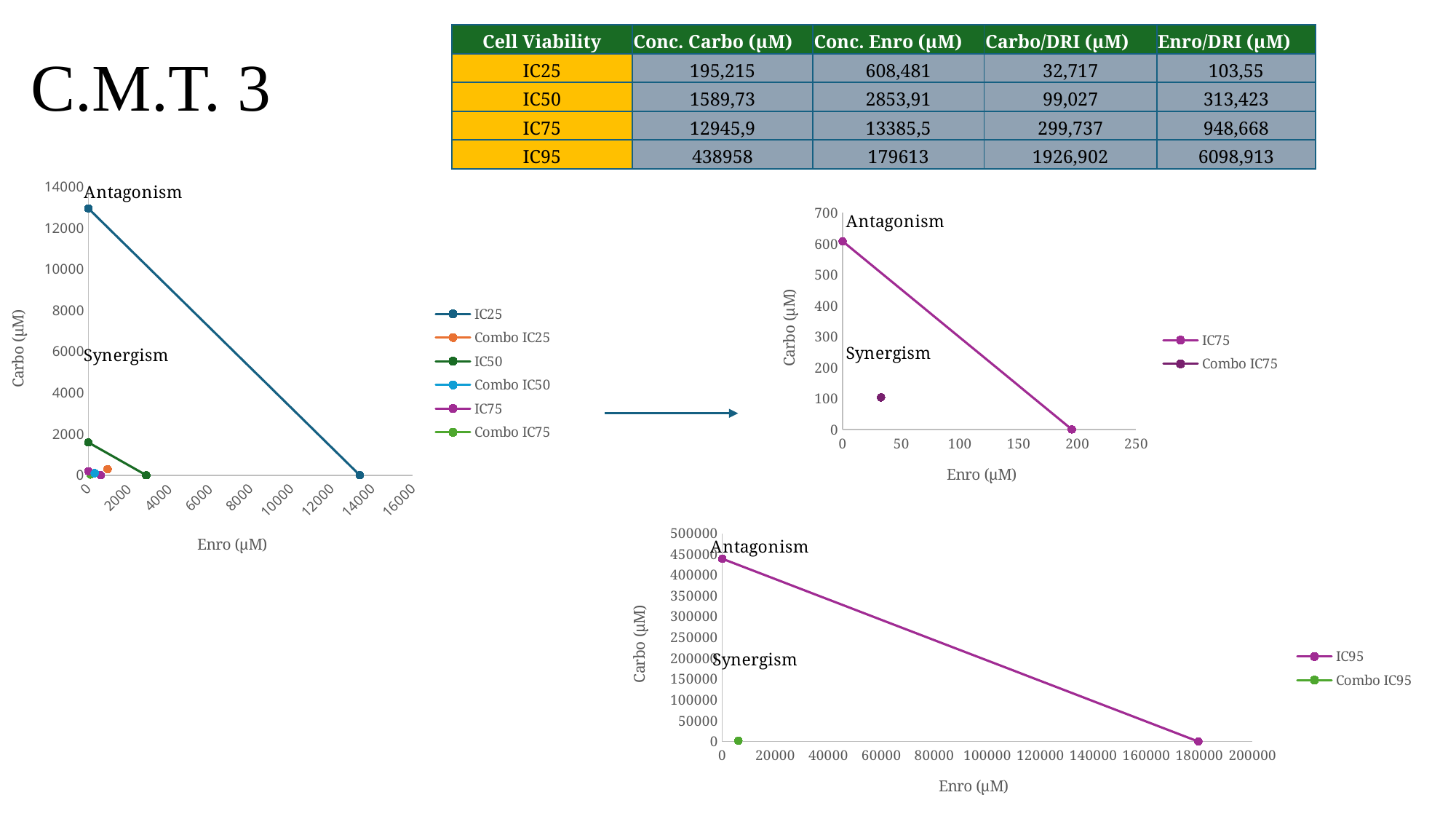

# C.M.T. 3
| Cell Viability | Conc. Carbo (µM) | Conc. Enro (µM) | Carbo/DRI (µM) | Enro/DRI (µM) |
| --- | --- | --- | --- | --- |
| IC25 | 195,215 | 608,481 | 32,717 | 103,55 |
| IC50 | 1589,73 | 2853,91 | 99,027 | 313,423 |
| IC75 | 12945,9 | 13385,5 | 299,737 | 948,668 |
| IC95 | 438958 | 179613 | 1926,902 | 6098,913 |
### Chart
| Category | | | | | | |
|---|---|---|---|---|---|---|
### Chart
| Category | | |
|---|---|---|
### Chart
| Category | | |
|---|---|---|

## Slide 4
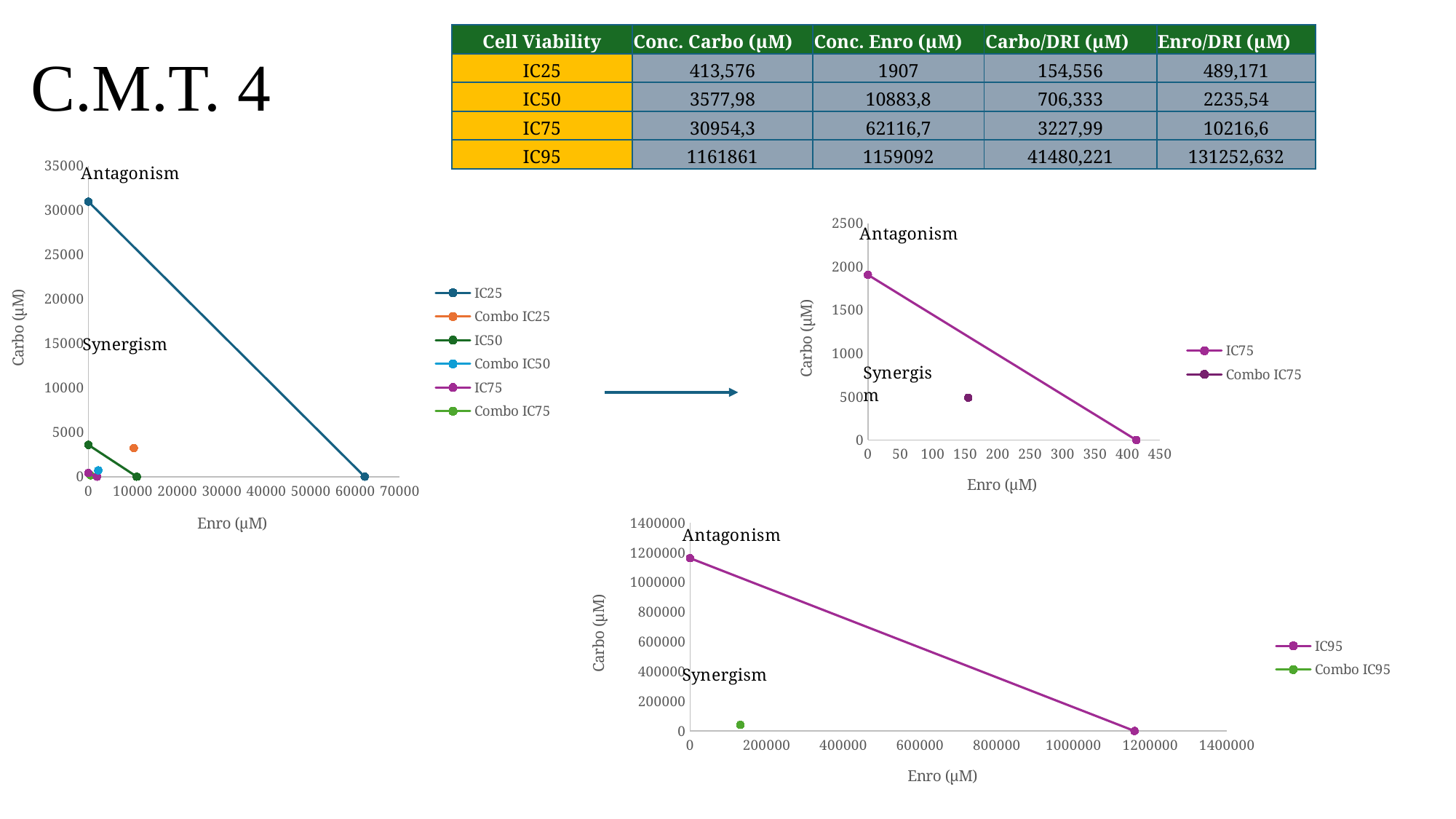

# C.M.T. 4
| Cell Viability | Conc. Carbo (µM) | Conc. Enro (µM) | Carbo/DRI (µM) | Enro/DRI (µM) |
| --- | --- | --- | --- | --- |
| IC25 | 413,576 | 1907 | 154,556 | 489,171 |
| IC50 | 3577,98 | 10883,8 | 706,333 | 2235,54 |
| IC75 | 30954,3 | 62116,7 | 3227,99 | 10216,6 |
| IC95 | 1161861 | 1159092 | 41480,221 | 131252,632 |
### Chart
| Category | | | | | | |
|---|---|---|---|---|---|---|
### Chart
| Category | | |
|---|---|---|
### Chart
| Category | | |
|---|---|---|

## Slide 5
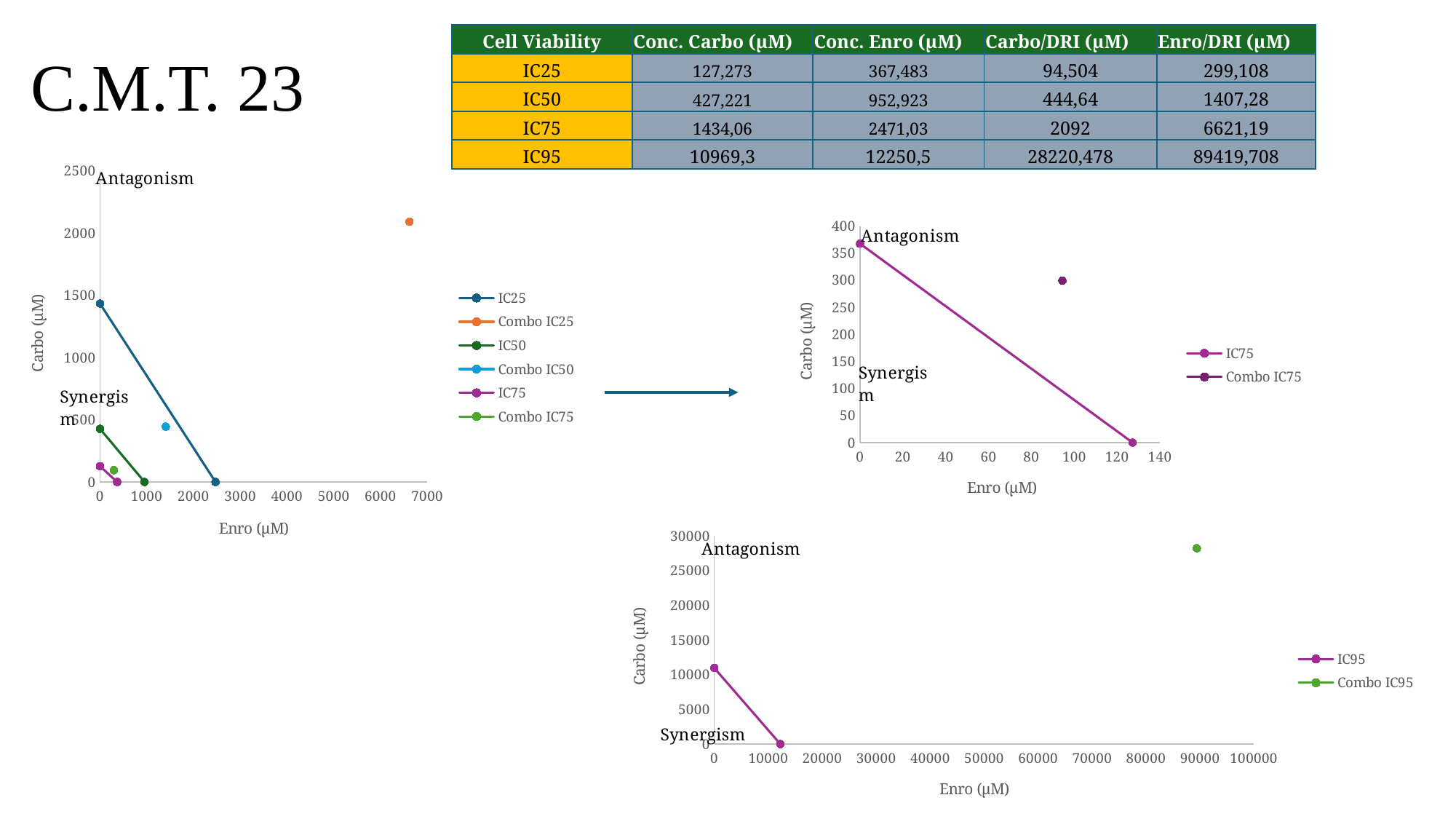

# C.M.T. 23
| Cell Viability | Conc. Carbo (µM) | Conc. Enro (µM) | Carbo/DRI (µM) | Enro/DRI (µM) |
| --- | --- | --- | --- | --- |
| IC25 | 127,273 | 367,483 | 94,504 | 299,108 |
| IC50 | 427,221 | 952,923 | 444,64 | 1407,28 |
| IC75 | 1434,06 | 2471,03 | 2092 | 6621,19 |
| IC95 | 10969,3 | 12250,5 | 28220,478 | 89419,708 |
### Chart
| Category | | | | | | |
|---|---|---|---|---|---|---|
### Chart
| Category | | |
|---|---|---|
### Chart
| Category | | |
|---|---|---|

## Slide 6
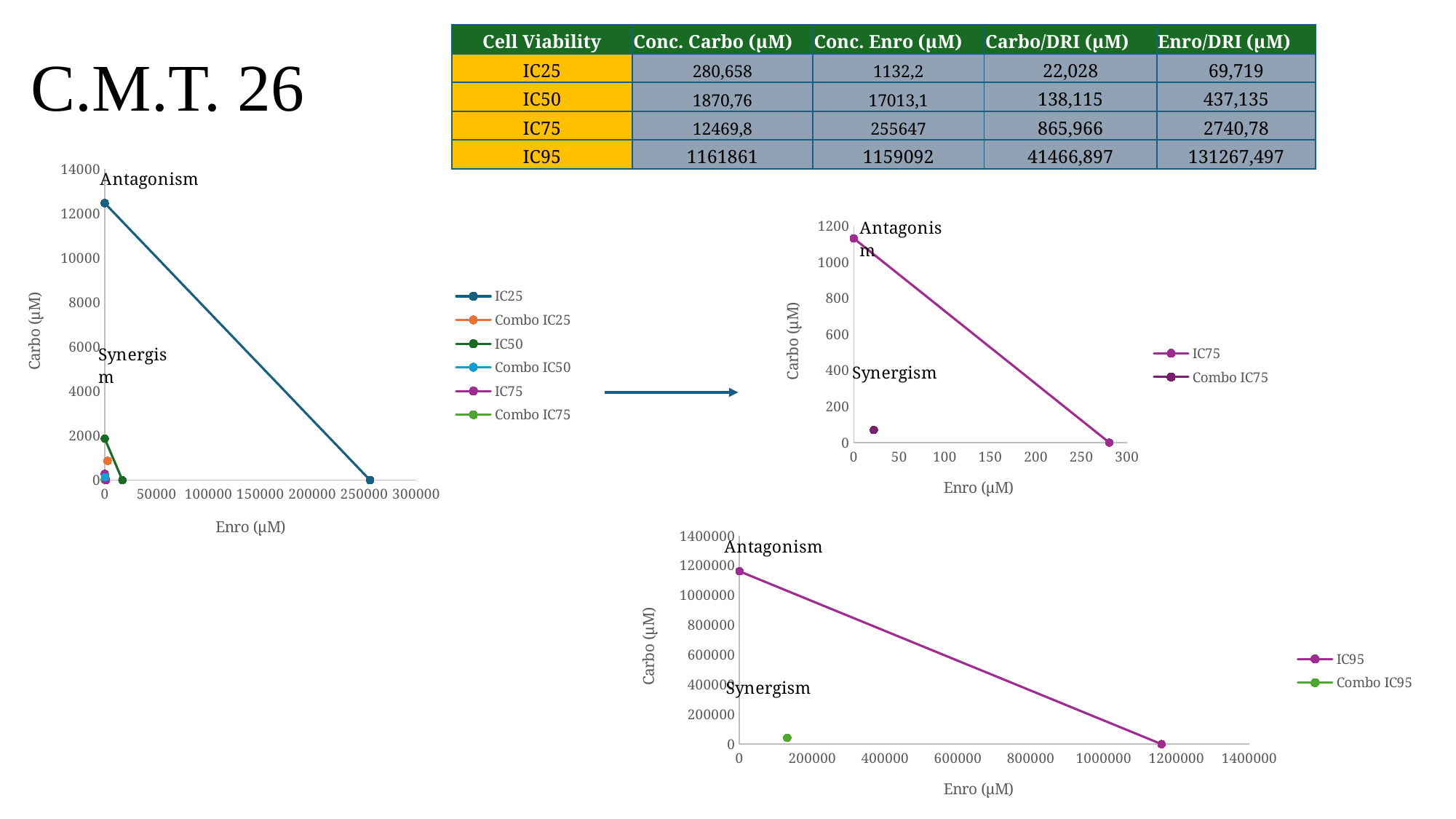

# C.M.T. 26
| Cell Viability | Conc. Carbo (µM) | Conc. Enro (µM) | Carbo/DRI (µM) | Enro/DRI (µM) |
| --- | --- | --- | --- | --- |
| IC25 | 280,658 | 1132,2 | 22,028 | 69,719 |
| IC50 | 1870,76 | 17013,1 | 138,115 | 437,135 |
| IC75 | 12469,8 | 255647 | 865,966 | 2740,78 |
| IC95 | 1161861 | 1159092 | 41466,897 | 131267,497 |
### Chart
| Category | | | | | | |
|---|---|---|---|---|---|---|
### Chart
| Category | | |
|---|---|---|
### Chart
| Category | | |
|---|---|---|

## Slide 7
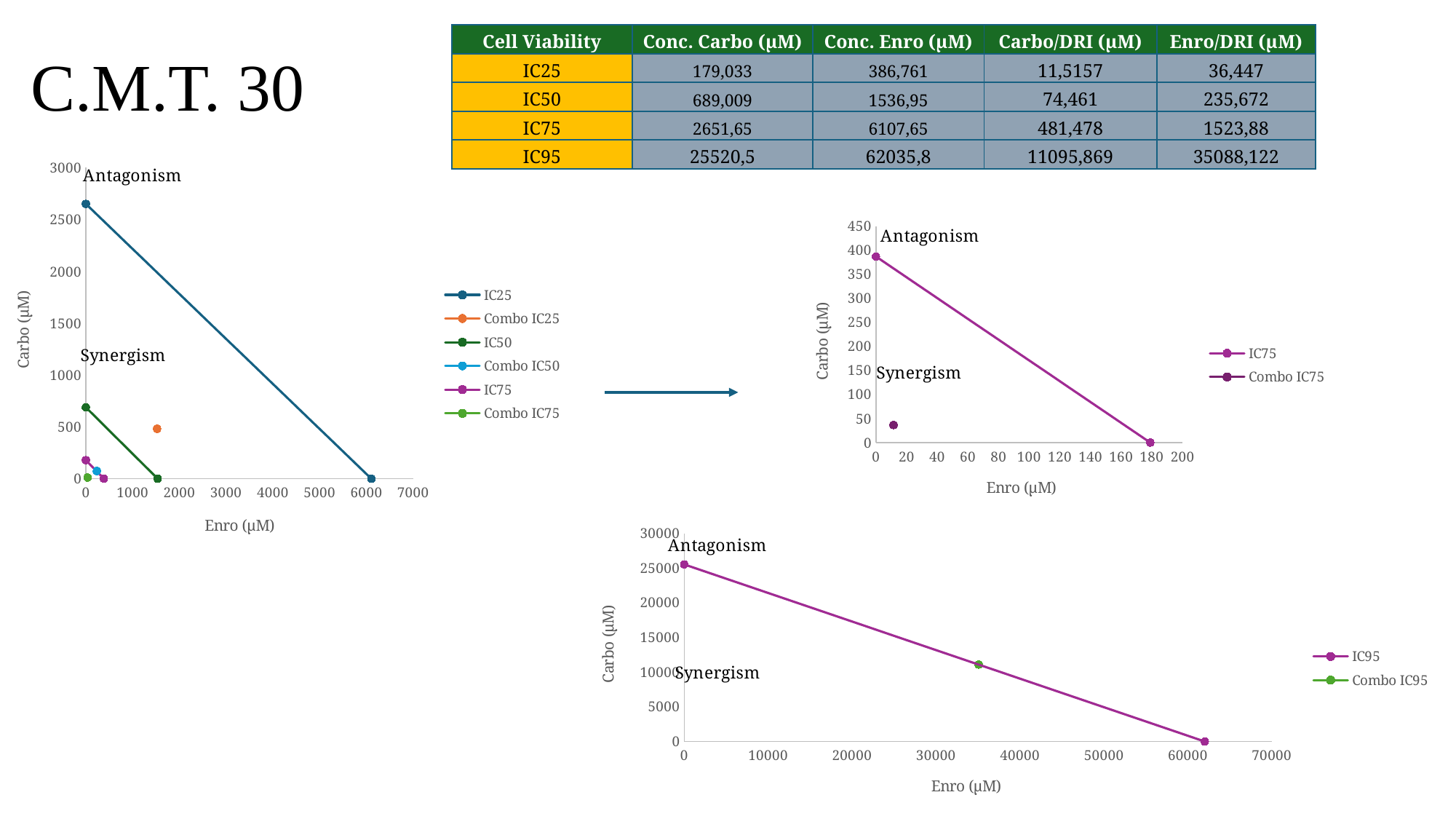

# C.M.T. 30
| Cell Viability | Conc. Carbo (µM) | Conc. Enro (µM) | Carbo/DRI (µM) | Enro/DRI (µM) |
| --- | --- | --- | --- | --- |
| IC25 | 179,033 | 386,761 | 11,5157 | 36,447 |
| IC50 | 689,009 | 1536,95 | 74,461 | 235,672 |
| IC75 | 2651,65 | 6107,65 | 481,478 | 1523,88 |
| IC95 | 25520,5 | 62035,8 | 11095,869 | 35088,122 |
### Chart
| Category | | | | | | |
|---|---|---|---|---|---|---|
### Chart
| Category | | |
|---|---|---|
### Chart
| Category | | |
|---|---|---|

## Slide 8
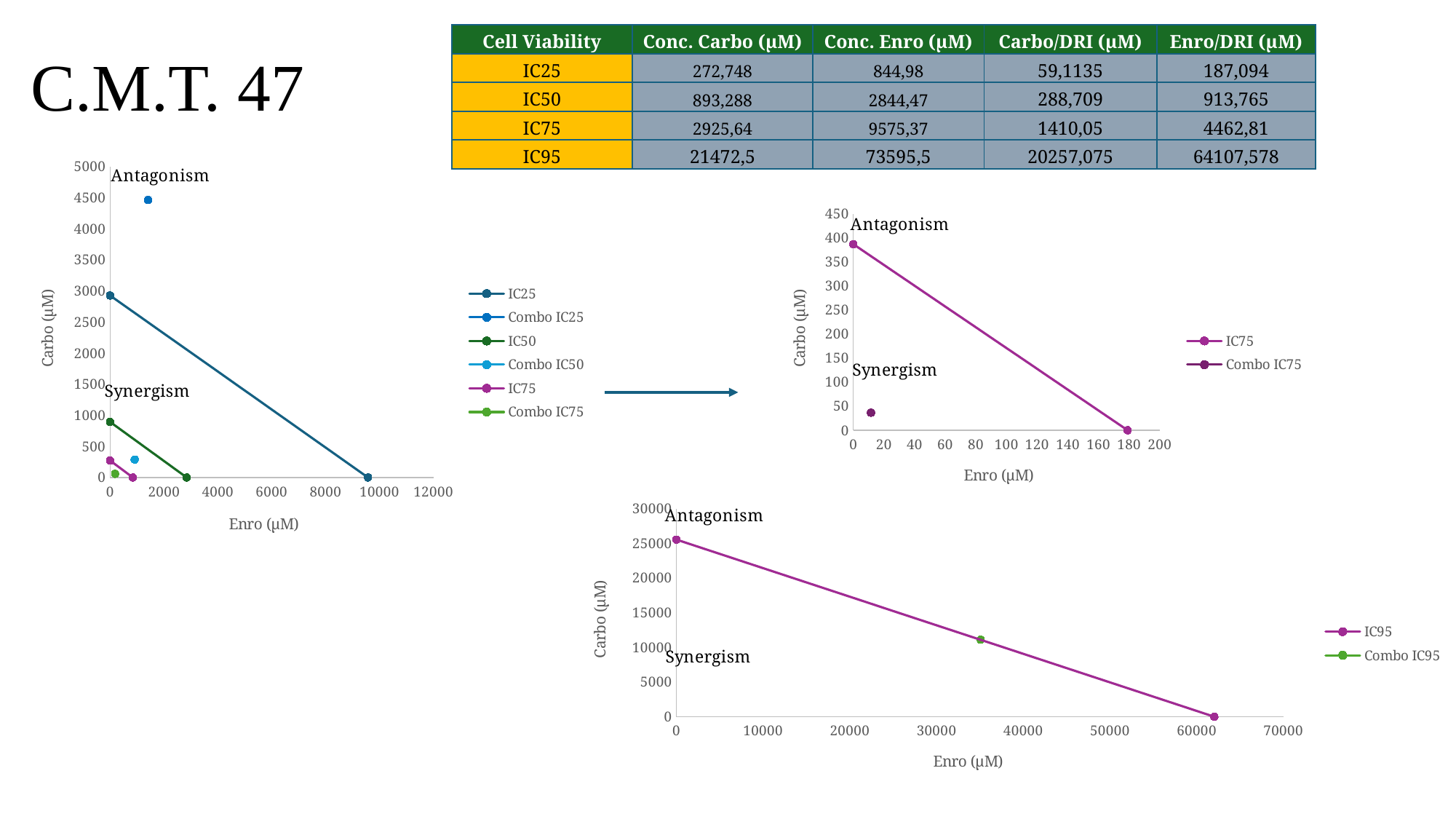

# C.M.T. 47
| Cell Viability | Conc. Carbo (µM) | Conc. Enro (µM) | Carbo/DRI (µM) | Enro/DRI (µM) |
| --- | --- | --- | --- | --- |
| IC25 | 272,748 | 844,98 | 59,1135 | 187,094 |
| IC50 | 893,288 | 2844,47 | 288,709 | 913,765 |
| IC75 | 2925,64 | 9575,37 | 1410,05 | 4462,81 |
| IC95 | 21472,5 | 73595,5 | 20257,075 | 64107,578 |
### Chart
| Category | | | | | | |
|---|---|---|---|---|---|---|
### Chart
| Category | | |
|---|---|---|
### Chart
| Category | | |
|---|---|---|

## Slide 9
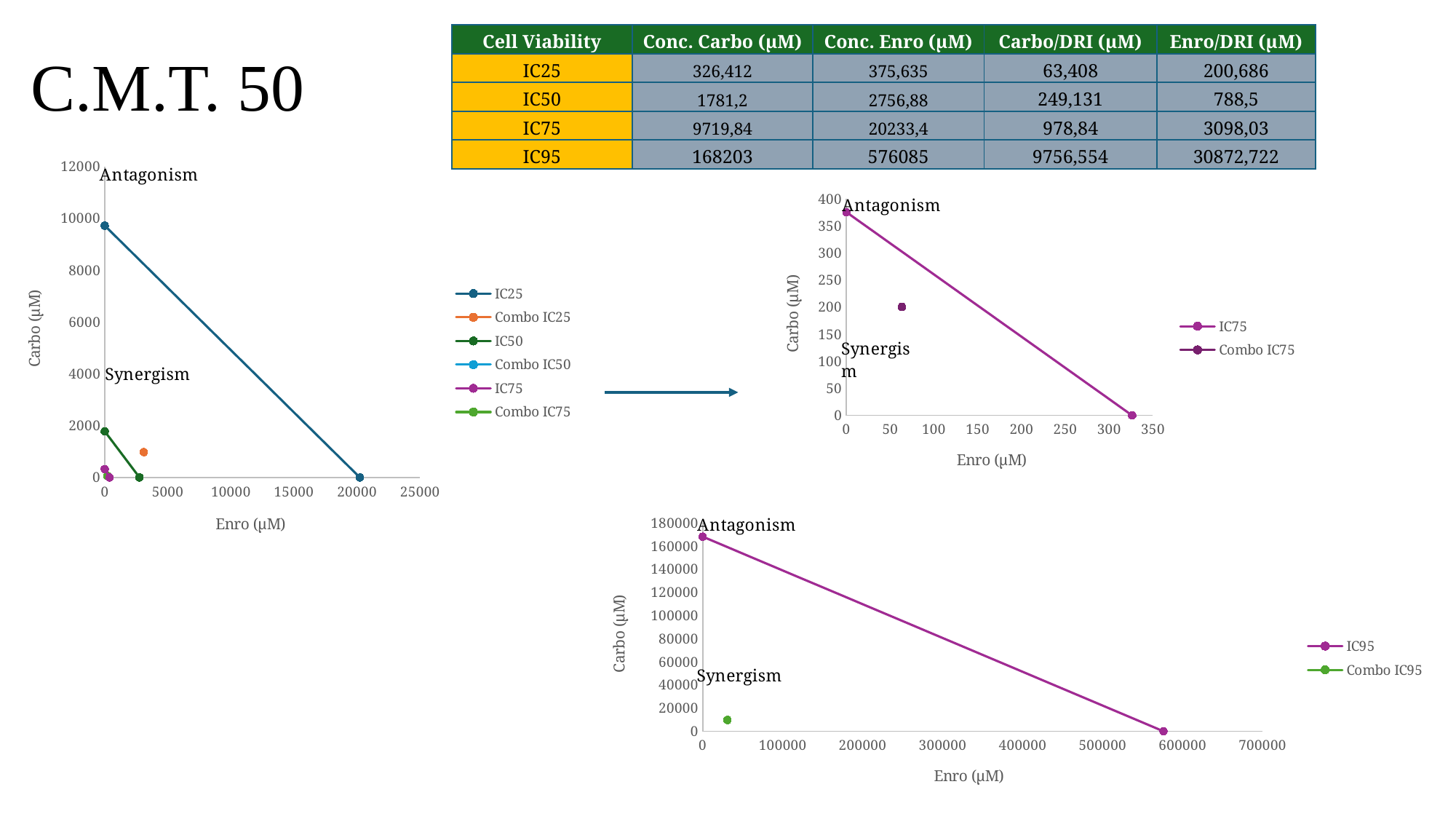

# C.M.T. 50
| Cell Viability | Conc. Carbo (µM) | Conc. Enro (µM) | Carbo/DRI (µM) | Enro/DRI (µM) |
| --- | --- | --- | --- | --- |
| IC25 | 326,412 | 375,635 | 63,408 | 200,686 |
| IC50 | 1781,2 | 2756,88 | 249,131 | 788,5 |
| IC75 | 9719,84 | 20233,4 | 978,84 | 3098,03 |
| IC95 | 168203 | 576085 | 9756,554 | 30872,722 |
### Chart
| Category | | | | | | |
|---|---|---|---|---|---|---|
### Chart
| Category | | |
|---|---|---|
### Chart
| Category | | |
|---|---|---|

## Slide 10
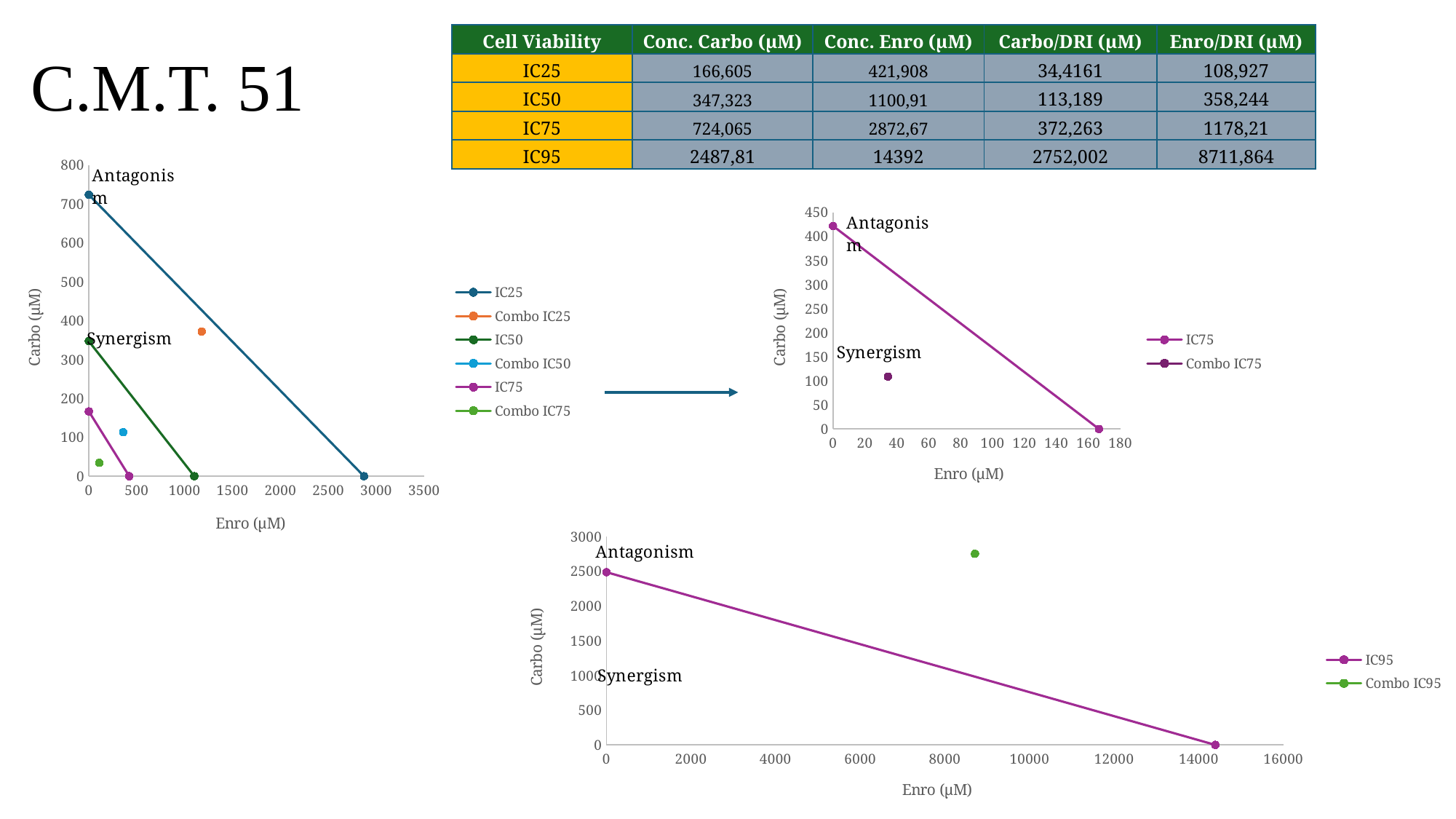

# C.M.T. 51
| Cell Viability | Conc. Carbo (µM) | Conc. Enro (µM) | Carbo/DRI (µM) | Enro/DRI (µM) |
| --- | --- | --- | --- | --- |
| IC25 | 166,605 | 421,908 | 34,4161 | 108,927 |
| IC50 | 347,323 | 1100,91 | 113,189 | 358,244 |
| IC75 | 724,065 | 2872,67 | 372,263 | 1178,21 |
| IC95 | 2487,81 | 14392 | 2752,002 | 8711,864 |
### Chart
| Category | | | | | | |
|---|---|---|---|---|---|---|
### Chart
| Category | | |
|---|---|---|
### Chart
| Category | | |
|---|---|---|
